# Thermochemical Treatment of Wastewater Residual Solids for Global Mitigation of Emerging Contaminants

**DOI:** 10.1038/s41467-026-74242-2

**Published:** 2026-06-12

**Authors:** Jianan Feng, Jeremy S. Guest

**Affiliations:** 1 The Grainger College of Engineering, Department of Civil and Environmental Engineering, University of Illinois Urbana-Champaign, Urbana, IL 61801 USA; 2 Institute for Sustainability, Energy, and Environment, University of Illinois Urbana-Champaign, Urbana, IL 61801 USA

**Keywords:** Chemical engineering, Pollution remediation, Water resources

## Abstract

Emerging contaminants are ubiquitous across the environment, posing rising ecological and public-health threats. Through a global data compilation, we show wastewater residual solids, by-products of wastewater treatment, represent a concentrated reservoir of emerging contaminants, capturing an estimated 20%, 24%, and 13% of global releases of microplastics, pharmaceuticals, and antibiotic resistance genes, respectively. This concentration creates a strategic intervention point where eliminating emerging contaminants within wastewater residual solids can substantially reduce environmental loading. Leveraging this opportunity requires next-generation technologies such as thermochemical processing capable of near-complete emerging contaminant destruction. Simulation-based evaluation suggests thermochemical wastewater residual solids management exhibits overlapping cost and greenhouse gas emission ranges with conventional systems, with average costs typically higher and average greenhouse gas emission consistently lower. The Global North faces higher costs yet greater investment capacity, whereas the Global South benefits economically but faces infrastructure gaps. Collectively, this analysis provides insights into intercepting emerging contaminants, reframing wastewater systems as active planetary defenses.

## Abstract (web summary)

The authors show that wastewater residual solids act as concentrated reservoirs of emerging contaminants, and that thermochemical treatment could enable their destruction while reducing environmental impacts, supporting global wastewater management transitions.

## Introduction

Synthetic chemicals underpin modern life but now pervade the global environment. Approximately 350,000 synthetic chemicals are registered for production and use worldwide, yet fewer than 1% are systematically regulated^1,2^. Many are persistent, bioaccumulative, and/or toxic, and are increasingly recognized as emerging contaminants (ECs)^3,4^. ECs—including nanoplastics, microplastics (MPs), pharmaceuticals (PhACs), antibiotic resistance genes (ARGs), and per- and polyfluoroalkyl substances (PFAS)—have been detected across all major environmental compartments, from agricultural soils and river sediments to drinking-water aquifers and oceans^5–21^. Global exposure assessments indicate populations worldwide encounter ECs daily through water, food, and air^22,23^. Their persistence and mobility have created a planetary-scale chemical burden whose complexity and diffuseness now rival those of identified Earth-system threats^24^.

Efforts to mitigate ECs remain fragmented and reactive. Source-control policies have successfully reduced emissions of well-characterized industrial compounds such as PFAS^25^. However, these measures fail to address the thousands of lesser-known or newly synthesized chemicals entering the environment through domestic use, agriculture, and waste mismanagement^26^. Once released, these contaminants disperse through hydrological, atmospheric, and biogeochemical pathways, forming diluted environmental reservoirs that are nearly impossible to treat^27–29^. The absence of scalable and globally implementable solutions underscores an urgent question: where in the global chemical cycle can ECs be intercepted most effectively before irreversible dispersal?

Water systems offer a promising yet underappreciated answer. A substantial fraction of anthropogenic chemicals eventually enters wastewater networks through washing, excretion, or surface runoff, which collectively channel contaminants into centralized water resource recovery facilities (WRRFs)^13,30,31^. Acting as the engineered “kidneys” of human societies, WRRFs now serve over one-third of the world’s population^32^. More importantly, they have the potential to capture a broad spectrum of pollutants into a smaller, more manageable reservoir—wastewater residual solids (WWRS)—while purifying water for release or reuse within established monitoring and regulatory frameworks, making them the most extensive human-made network for intercepting chemical pollutants^30,31,33–37^. This concentration effect transforms wastewater systems from passive conduits into strategic interception points in the global EC cycle. However, current WWRS management practices, which are dominated by stabilization and disposal, cannot effectively mitigate or destroy ECs, leading to EC reintroduction into the environment via landfilling^38^ or land application^39^. This technological limitation creates a critical need for a new class of treatment processes capable of achieving molecular-level destruction.

Elevated temperature offers one of the most reliable mechanisms for breaking down persistent chemical structures. Harnessing this principle, thermochemical processes—such as hydrothermal liquefaction (HTL), hydrothermal alkaline treatment (HALT), supercritical water oxidation (SCWO), pyrolysis, and gasification—typically operate at 250 °C to 900 °C, offering a promising route to achieve near-complete destruction of ECs^40–54^. Simultaneously, these technologies have the potential to reduce pathogen loads and residual volumes, mitigate greenhouse gas (GHG) emissions, and generate renewable energy, thereby delivering synergistic environmental and economic benefits^55–59^. Importantly, many of these technologies can be integrated into existing WRRFs, enabling scalable and distributed deployment.

Here, we demonstrate that targeting WWRS can transform wastewater infrastructure into an active global defense against ECs. We first analyzed global datasets to quantify EC accumulation in WWRS relative to other environmental compartments, demonstrating WWRS as one of the planet’s most concentrated EC reservoirs. We then examined the operational conditions, removal efficiencies, and underlying mechanisms of representative thermochemical technologies, highlighting their potential for near-complete EC destruction. Finally, we performed a simulation-based evaluation of country-level (henceforth, “country” refers to both countries and territories) costs, GHG emissions, and transition opportunities to identify priority regions where the integration of thermochemical treatment can maximize economic and environmental benefits. These findings are intended to inform national- and global-scale prioritization of WWRS management strategies, while site-specific design and implementation decisions require further facility-level evaluation. In this context, wastewater systems can be reframed as planetary chemical filters rather than endpoints of waste disposal, providing insights into managing a growing burden facing communities across the globe. Just as the 20^th^-century sanitation revolutionized pathogen control^60^, 21^st^-century wastewater systems can evolve into a coordinated global infrastructure for mitigating ECs, defending both ecological and public health.

## Results

### Emerging contaminants in Wastewater Residual Solids

**Fig. 1:**
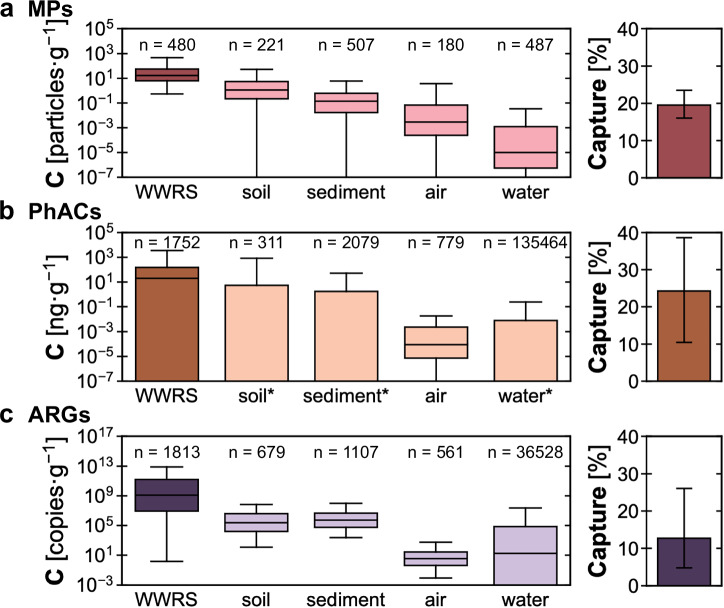
Global concentrations and distributions of representative emerging contaminants (ECs). **a** microplastics (MPs, red), **b** pharmaceuticals (PhACs, orange), and **c** antibiotic resistance genes (ARGs, purple). The left panels show EC concentrations in wastewater residual solids (WWRS; darker color scheme) compared to other environmental compartments (lighter color scheme). Concentrations of EC in WWRS, soil, and sediment are on a dry-weight basis. Whiskers, boxes, and midlines of box plots represent 5th and 95th percentiles, 25th and 75th percentiles, and 50th percentile, respectively. A star symbol beside a compartment name indicates a median concentration of zero. The right panel shows the percentage of each EC released to the environment that could be captured by WWRS, with error bars representing the 5th to 95th percentile range. Source data are provided as a Source Data file.

As by-products of engineered wastewater treatment systems, WWRS represent a controllable, concentrated, and cross-contaminant point for the elimination of global ECs. WWRS concentrate ECs from diverse anthropogenic and diffuse sources, acting as a barrier that prevents uncontrolled dispersal into the environment. Across all major EC classes, concentrations in WWRS can be orders of magnitude higher than in other environmental compartments (Fig. 1, left panel). Specifically, MPs are present at 17 [0.54 to 470] particles·g^−1^ (median followed by 5^th^ and 95^th^ percentiles in brackets; concentrations in solids reported on a dry-weight basis) in WWRS, compared with 1.1 [0 to 53] particles·g^−1^ in soil, 0.14 [0 to 6.0] particles·g^−1^ in sediment, 0.0029 [0 to 3.7] particles·g^−1^ in air, and 1.0 × 10^−5^ [2.2 × 10^−10^ to 0.034] particles·g^−1^ in water. This enrichment reflects both the strong sorption affinity and density-driven settling of MPs during wastewater treatment. For PhACs, concentrations span a wider range due to compound-specific solubility, degradation kinetics, and surface interactions. Median concentrations in soil, sediment, and water are effectively zero, and reported concentrations in air are only 9.0 × 10^−5^ [0 to 0.019] ng·g^−1^. In contrast, PhACs concentrations in WWRS reach 20 [0 to 3500] ng·g^−1^, with the median value far exceeding those in natural environments. ARGs exhibit a similar pattern, with concentrations (ranked by median values) of 1.2 × 10^9^ [1.5 to 7.9 × 10^12^] copies·g^−1^ in WWRS, 5.3 × 10^5^ [2300 to 1.0×10^8^] copies·g^−1^ in sediment, 2.4 × 10^5^ [120 to 6.8 × 10^7^] copies·g^−1^ in soil, 18 [0 to 2.3 × 10^7^] copies·g^−1^ in water, and 3.4 [0.0082 to 550] copies·g^−1^ in air. Complementary analyses of perfluorooctanoic acid (PFOA), perfluorooctane sulfonate (PFOS), and legacy persistent organic pollutants (polybrominated diphenyl ethers, polychlorinated biphenyls, and polychlorinated dibenzo-p-dioxins and polychlorinated dibenzofurans) similarly identify WWRS as a highly concentrated global reservoir (Fig. S1, where “S” indicates Supplementary Information). Collectively, these datasets confirm that WWRS is a cross-contaminant sink, where multiple classes of ECs converge at levels sufficient to enable centralized intervention.

To evaluate the global significance of this containment role, three representative ECs—MPs, PhACs, and ARGs—were selected for quantitative assessment based on data availability and environmental relevance. These groups collectively capture the physical, chemical, and biological diversity of EC behavior. Specifically, MPs exemplify persistent particulates that accumulate through mechanical and hydrodynamic pathways; PhACs represent dissolved and sorptive organics governed by molecular transport and transformation; and ARGs represent biological contaminants that propagate through horizontal gene transfer and microbial adaptation under anthropogenic pressure. Together, they provide a first-order yet comprehensive representation of EC accumulation, persistence, and potential elimination within WWRS.

Global transmission modeling of these three ECs reveals that WWRS capture a substantial share of total emissions across environmental pathways (Fig. 1, right panel). For MPs, six major sources were considered (Table S1). MPs from synthetic textiles and personal care products enter WRRFs directly via sewers, while those from vehicle tires, road markings, and city dust contribute via runoff in regions with combined sewer systems^61^. As a result, the model estimated 20% [16% to 24%] of total global MP emissions migrate through WWRS. For PhACs, emissions originate from both excreted residues and disposal through trashing and flushing (Table S3)^62,63^. Overall, 24% [10% to 39%] of global PhACs are routed through WWRS. For ARGs, whose primary transmission pathway is animal excreta^64^, transmission fluxes were approximated using antibiotic usage as a proxy due to their strong correlation (Table S5)^12^. Results indicated 13% [4.7% to 26%] of ARGs globally pass through WWRS. These results highlight WWRS as a globally significant intervention point capable of simultaneously addressing multiple EC categories at meaningful scales. The relatively consistent capture fractions across uncertainty ranges (Section S1) also suggest that these system-level conclusions are robust, although the exact magnitudes remain sensitive to assumptions regarding emission sources and transport pathways. It is important to note, however, WWRS are not the dominant sink for all ECs. For example, while PFAS remain a high-priority target due to their persistence and toxicity, the majority (e.g., around 90%) of PFAS emissions arise directly from industrial point sources rather than municipal systems^65^. Estimates further suggest that contributions routed through WWRS account for only about 0.1% of total emissions of PFOA and PFOS (Section S1.5)^65^, indicating that PFAS removal within WWRS is unlikely to substantially influence the global PFAS mass balance relative to upstream source-control strategies. However, as regulatory bans and industrial phase-outs take effect, WWRS will continue to act as a long-term containment and treatment point for residual PFAS and other ECs.

### Thermochemical technologies for efficient EC destruction

**Fig. 2:**
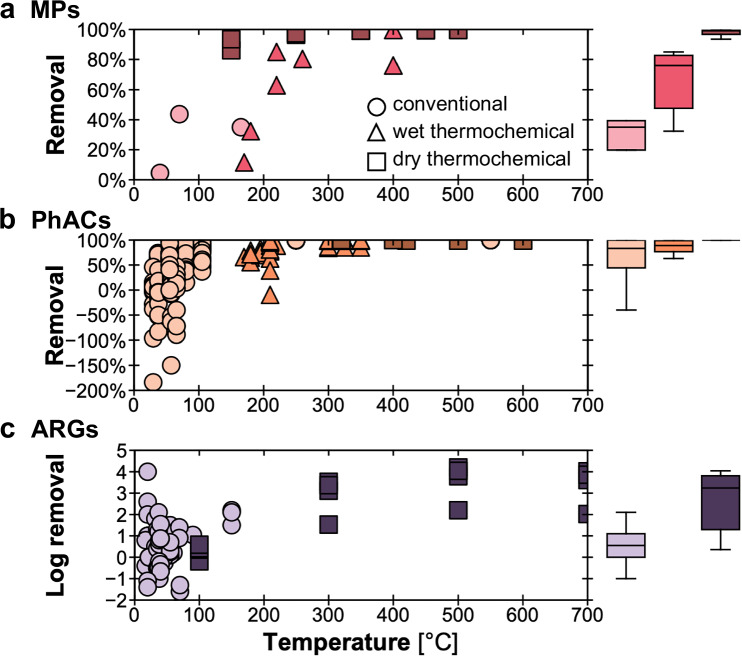
Removal rate of representative emerging contaminants during wastewater residual solids management. **a** microplastics (MPs, red), **b** pharmaceuticals (PhACs, orange), and **c** antibiotic resistance genes (ARGs, purple). Circles with light fill, triangles with moderate fill, and squares with dark fill represent conventional, wet thermochemical, and dry thermochemical technologies, respectively. Whiskers, boxes, and midlines of box plots represent 5th and 95th percentiles, 25th and 75th percentiles, and 50th percentile, respectively. Source data are provided as a Source Data file.

Thermochemical treatment of WWRS presents an opportunity to couple WWRS valorization with EC destruction. Broadly, WWRS treatment technologies can be categorized as conventional (e.g., digestion, composting), wet thermochemical (e.g., hydrothermal carbonization [HTC], HTL), and dry thermochemical (e.g., pyrolysis, gasification). These groups differ in reaction pathways, operational temperatures, and oxidation potentials, which collectively control EC removal. Across all classes of ECs analyzed, removal efficiency generally increases with temperature, reaching a plateau as conditions approach the thermochemical range (Fig. 2). Below, three representative EC classes—MPs, PhACs, and ARGs—are examined to illustrate treatment performance across technologies.

#### Microplastics (MPs)

MPs are chemically stable and persist through conventional WWRS management systems, where removal relies on mild thermal or microbial processes (e.g., anaerobic digestion, composting)^33,66^, achieving only modest reductions ( < 50%; Fig. 2a). Wet thermochemical processes markedly enhance MP breakdown through polymer depolymerization, β-scission, and radical oxidation^67^. Reaction severity, hydrolysis, and partial oxidation intensify with temperature^68,69^, increasing the average MP removal from 59% under HTC (180 °C–250 °C) to 88% under HTL (250 °C–400 °C). Dry thermochemical processes at 150 °C–500 °C can achieve higher MP removal (99% [89%–100%]), possibly owing to rapid radical generation^70^. Collectively, these findings suggest thermochemical treatment of WWRS could be an effective substitute for conventional approaches, especially in contexts where MP contamination and land application of biosolids are prevalent.

#### Pharmaceuticals (PhACs)

PhACs are thermally labile, with decomposition typically occurring around 200 °C–300 °C^71^. Conventional systems depend on adsorption, biodegradation, or low-temperature chemical oxidation, resulting in wide removal efficiency (83% [−49% to 100%]; Fig. 2b), with negative values indicating net increase which may be due to deconjugation of PhACs metabolites^72^. Wet thermochemical processes at 170 °C–350 °C achieve substantially higher degradation (89% [59%–100%]) through hydrolysis, deamination, and cyclization, with increasing temperatures driving the transition from ionic to free radical reactions^43^. Dry thermochemical systems at 320 °C–600 °C further amplify radical generation^70^, facilitating near-complete removal of PhACs. These findings highlight the capacity of thermochemical treatment to eliminate PhAC residuals from WWRS at scale.

#### Antibiotic resistance genes (ARGs)

ARGs persist across conventional WWRS treatment processes, including thermal hydrolysis and digestion, which only partially deactivate genetic material or leave residual fragments capable of horizontal gene transfer, leading to the propagation of ARGs^73,74^. The overall log-removal of ARGs under conventional treatment is 0.55 [−1.0–2.1] (Fig. 2c). On the other hand, since rapid DNA degradation can happen between 100 °C and 200 °C^75^, thermochemical processes provide the necessary thermal and oxidative conditions for irreversible nucleic acid breakdown. In particular, dry thermochemical technologies at 100 °C–700 °C achieve near-complete DNA mineralization and rapid ARG elimination at elevated temperatures. Data for wet thermochemical systems remain scarce, largely because near-total degradation precludes analytical detection. Nonetheless, current evidence indicates that wet hydrothermal processing can substantially reduce the dissemination of ARGs from other organic wastes, including residues generated during antibiotic manufacturing^76–78^.

Across EC classes, removal efficiency follows the general ascending trend of conventional, wet thermochemical, and dry thermochemical technologies. Removal mechanisms evolve from adsorption and physical capture under low temperature, to hydrolytic cleavage under moderate temperature, to radical-driven oxidation and mineralization under high temperature conditions. In practice, technology selection depends on WWRS properties (e.g., moisture content, biochemical compositions), discharge standards, and target contaminant profiles. Yet the consistently superior performance of thermochemical systems—coupled with their compatibility with resource recovery—positions them as scalable, globally deployable solutions for EC mitigation within WRRFs networks.

### Global implications of thermochemical WWRS management

Though thermochemical technologies demonstrate strong potential for mitigating ECs, their large-scale deployment further depends on financial feasibility, infrastructure readiness, and policy support. To elucidate these interdependencies, a techno-economic and environmental baseline was established for 105 countries to assess the global transition from conventional to thermochemical WWRS management. Due to limited facility-level configuration data, 25 representative conventional and 15 representative thermochemical systems (Table S10) were applied to estimate country-level cost and carbon intensity (CI). The analysis was limited to facilities with estimated WWRS production higher than 5 dry tonne·day^−1^, focusing on facilities where capital-intensive thermochemical technologies are most feasible^59^. While these estimates do not capture the full heterogeneity of individual WRRFs, they enable consistent cross-country comparisons of cost and emission changes associated with adopting thermochemical WWRS management.

**Fig. 3:**
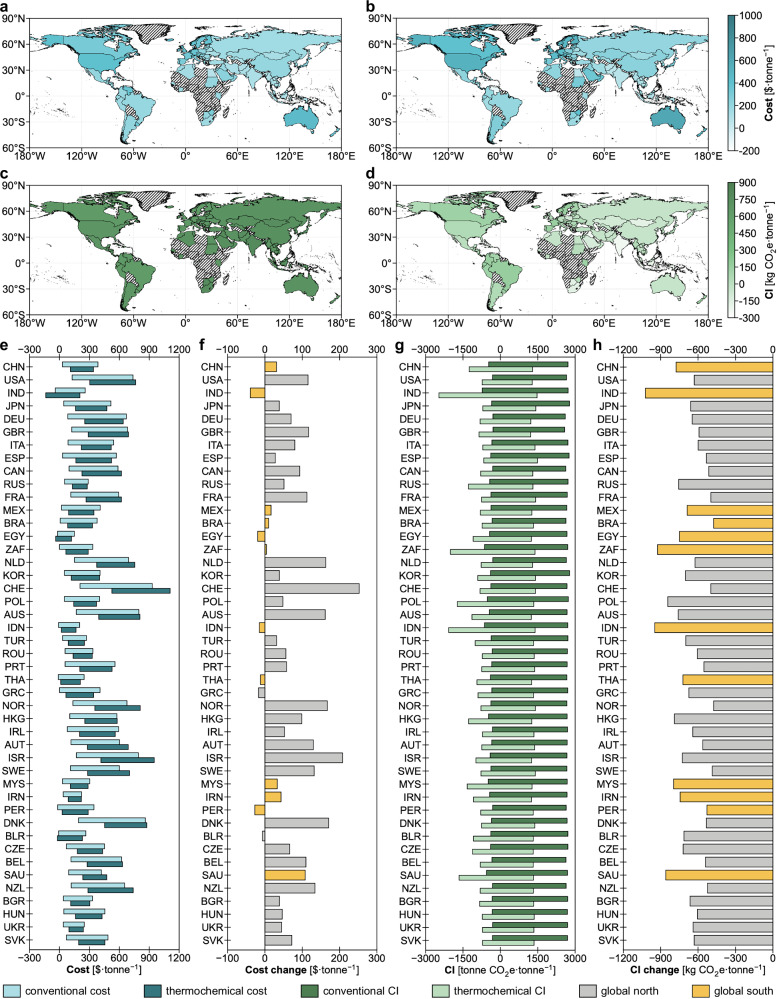
Country-level cost and carbon intensity (CI) of conventional and thermochemical wastewater residual solids management. Average country-level cost for **a** conventional and **b** thermochemical systems are shown in blue. Average country-level CI for **c** conventional and **d** thermochemical systems are shown in green. Darker colors in (**a**–**d**) indicate higher values. Hatched regions in (**a**–**d**) indicate area where data were unavailable or insufficient for analysis. **e** Estimated cost ranges of conventional (light blue) and thermochemical (darker blue) systems are shown for 45 countries, with the average change between the two shown in (**f**). **g** Estimated CI ranges of conventional (darker green) and thermochemical (lighter green) systems are shown for 45 countries, with the average change between the two shown in (**h**). Yellow and gray bars in (**f**) and (**h**) represent the Global North and Global South, respectively. Countries in (**e**–**h**) include those with more than 10 water resource recovery facilities in the analysis and are ranked from top to bottom in descending order by the number of WRRFs. Source data are provided as a Source Data file.

From a financial perspective, across all countries, average costs span from 65 to 580 $·tonne^−1^ for conventional systems and from −120 to 910 $·tonne^−1^ for thermochemical systems (Fig. 3a, b). Most countries would, on average, incur additional costs when shifting from conventional to thermochemical systems, though substantial overlaps exist between the cost ranges of both (Fig. 3e). This overlap is driven largely by economies of scale, as larger facilities can rapidly reduce the normalized cost ($·tonne^−1^) of thermochemical systems. The largest increases of average cost were observed in high-income Global North countries in North America, Western Europe, and Oceania, with an average increase of 94 $·tonne^−1^ above already elevated conventional baselines (Fig. 3f). These increases primarily reflect higher construction, chemical, and labor costs in these countries (Supplementary Data 1). Yet these same regions generally possess the fiscal capacity and regulatory maturity to invest in sustainability-oriented infrastructure, positioning them as potential first movers in the global transition^79^. Conversely, many low- and middle-income Global South countries exhibit modest average cost increases or even net savings (Fig. 3f). For example, India, Egypt, Peru, Thailand, and Indonesia demonstrate cost reductions between 12 and 39 $·tonne^−1^ relative to their conventional baselines, indicating intrinsic financial incentives for a transition to thermochemical systems. Moreover, because many Global South countries are still expanding wastewater treatment capacity, thermochemical systems could be directly integrated into new facilities, avoiding costly retrofits that often hinder technology adoption in the Global North. However, limited infrastructure, policy frameworks, and institutional capacity remain major challenges for large-scale deployment in the Global South^79^. It should be noted all costs reported here are based on nth-of-a-kind assumptions, which excluded the uncertainties and risks associated with first-of-a-kind facilities, whose costs can be significantly higher (Supplementary Data 1).

Beyond financial considerations, the transition to thermochemical systems yields notable environmental benefits, particularly in reducing average GHG emissions (Fig. 3c, d, g). Across analyzed countries, net reductions in average CI range from 460 to 1000 kg CO_2_e·tonne^−1^ (Fig. 3h), largely driven by the displacement of fossil fuels through recovered biofuels, long-term carbon sequestration via hydrochar and biochar, and the avoidance of direct emissions associated with conventional WWRS management (e.g., methane generation from landfilling). These co-benefits create opportunities for cost parity between thermochemical and conventional systems through carbon credit integration. However, the extent of this opportunity varies regionally, from negative (i.e., thermochemical systems are financially favorable without carbon credits) in several Asian, African, and Latin American countries to required incentives exceeding 500 $·tonne CO_2_e^−1^ in parts of Europe (Fig. S2). Current carbon credit values (0.1–160 $·tonne CO_2_e^−1^)^80^ are far below the parity threshold in high-cost economies, implying additional policy instruments—such as targeted subsidies, green bonds, or green infrastructure funds—will be necessary to catalyze deployment. In contrast, in low- and middle-income countries where parity could be reached below prevailing carbon prices, deployment of thermochemical WWRS management could be accelerated through integration into existing emission trading schemes or carbon offset programs. While many developing countries may lack mature domestic carbon markets or institutional frameworks^81^, private companies and international investors, often from high-income countries, already fund mitigation and carbon-reduction projects in these regions^82^. In the voluntary carbon-credit market, for example, a substantial fraction of carbon offset projects are based in developing countries and financed by external actors^83^. Moreover, international climate-finance mechanisms and multilateral development banks can channel capital to these countries to support clean-technology deployment^84–86^. Therefore, the economic and institutional gaps in many Global South regions do not necessarily preclude deployment, provided that appropriate external financing, technology transfer, and supportive policy frameworks are mobilized. By strategically leveraging such global flows, blended finance, and collaborative investment, thermochemical WWRS management can be scaled across the Global South, thereby contributing to broader global EC mitigation efforts.

## Discussion

The results of this study should be interpreted as first-order estimates of the global transition from conventional to thermochemical WWRS management systems as a promising pathway for mitigating ECs, reflecting a system-level assessment based on representative configurations and intended to capture directional trends rather than provide precise predictions for individual WRRFs. Accordingly, the results are most applicable to national- and global-scale prioritization, while facility-level implementation requires site-specific evaluation. A more systematic technical assessment of thermochemical systems for EC destruction—including reaction kinetics and scalability—remains a key area requiring future research. Detailed decision-making processes at multiple governance levels also warrant further investigation. Several additional limitations in this study could be addressed in future research to enhance analytical accuracy and policy relevance. First, although efforts were made to incorporate detailed datasets, available data remain insufficiently resolved. For example, due to limited information on current WWRS management practices, averages of representative system configurations were used to estimate costs and environmental impacts across countries. Moreover, while country-level data were employed, cost adjustments were primarily based on indices (e.g., price level index for construction and materials, income-based scaling factors for labor costs) rather than market-based data. Second, the TEA and LCA were conducted under steady-state assumptions, without explicitly accounting for the temporal dynamics of technology learning and deployment. Consequently, transition trajectories from conventional to thermochemical systems remain uncertain. Lastly, although the analysis benchmarked the performance of thermochemical WWRS management, it did not include facility-level uncertainty quantification, nor did it consider the possibility of centralized thermochemical hubs for shared WWRS processing across multiple WRRFs^59^. Overall, these limitations highlight the importance of advancing large-scale technology demonstrations, developing regionally resolved datasets, and integrating dynamic, time-explicit, and uncertainty- and scenario-informed modeling frameworks to accelerate the global transition toward thermochemical WWRS management as a resilient and sustainable strategy for EC mitigation.

On the other hand, this study advances a paradigm in which thermochemical WWRS management repositions wastewater systems as active, engineered interfaces between human and planetary metabolism. By intercepting a substantial fraction of the most concerning ECs before they disperse into soils, waters, and biota, this paradigm reimagines WWRS management as a deliberate, centralized barrier to chemical pollution—analogous to the transformative role of wastewater treatment in pathogen control a century ago. Embedding this paradigm within the circular economy framework integrates environmental remediation into productive infrastructure rather than treating it as an ancillary. Achieving this integration, however, requires cross-sectoral coordination: aligning waste and energy markets, recognizing contaminant destruction as a verifiable environmental service, and adapting regulatory frameworks for incentives. Without such institutional alignment, the potential of thermochemical technologies for EC elimination and resource circularity may remain under-realized.

TEA and LCA reveal that another barrier to large-scale deployment of thermochemical WWRS management is socio-economic. While average costs of thermochemical systems typically exceed those of conventional systems, the environmental benefits, including substantial reductions in greenhouse gas emissions, are greater. Incorporating these benefits in valuation frameworks through carbon markets, green infrastructure credits, or pollution accounting mechanisms, could catalyze financing and incentivize adoption. Incorporating EC destruction metrics into national pollution inventories and climate strategies would further reinforce the role of thermochemical WWRS management within integrated sustainability governance, transforming it from a compliance-driven activity into a measurable environmental service.

Ultimately, achieving a global impact in emerging contaminant mitigation will require equitable implementation across regions. The Global North, with established wastewater networks, regulatory frameworks, and investment capacity, is well-positioned to pioneer early deployment, accelerate technology learning, and drive down costs through standardization. In contrast, the Global South—where most future wastewater infrastructure will be built—offers the greatest opportunity for transformative impact, provided that cost-scalable thermochemical systems can be deployed alongside expanding sanitation networks. Strategic international partnerships, through climate finance, multilateral development banks, and technology transfer initiatives, are essential to overcome financing and institutional gaps. Reconceiving WWRS as a planetary contaminant sink therefore reframes wastewater management from local sanitation services to a global chemical governance instrument. When implemented equitably, the paradigm shift to thermochemical WWRS management could become an important tool for restoring the chemical balance of the biosphere.

## Methods

### Global emerging contaminant distribution

A comprehensive literature review was conducted to compile concentration data for major classes of emerging contaminants (ECs), including microplastics (MPs), pharmaceuticals (PhACs), antibiotic resistance genes (ARGs), per- and polyfluoroalkyl substances (PFAS), and three legacy persistent organic pollutants (polychlorinated biphenyls, polybrominated diphenyl ethers, and polychlorinated dibenzo-*p*-dioxins and polychlorinated dibenzofurans). Data were collected across wastewater residual solids (WWRS) and four environmental compartments (soil, sediment, air, and water). The literature search was performed using Google Scholar with the query structures combining contaminant classes and environmental compartments (e.g., microplastics concentration in wastewater residual solids). For WWRS, soil, and sediment, only measurements explicitly reported on a dry-weight basis were included to ensure comparability. All concentrations were standardized to a *per gram* basis, assuming densities of 1000 kg·m^−3^ for water and 1.225 kg·m^−3^ for air. For PhACs, ARGs, and legacy persistent organic pollutants, both individual compound concentrations and aggregated values were extracted to accommodate varying reporting formats. This discrepancy was considered to be randomly distributed across compartments and was therefore expected to have minimal impact on the relative magnitudes observed. Additionally, the data collection did not explicitly distinguish between primary and secondary sources, which may introduce some duplication. However, even conservatively removing all identical values (thereby also eliminating distinct data points sharing the same reported concentration) does not alter relative patterns in Fig. 1 and Fig. S1 (Fig. S3). The only exception is for polychlorinated dibenzo-*p*-dioxins and polychlorinated dibenzofurans, whose median concentration in WWRS becomes slightly lower than that in soil and sediment after this removal. In total, approximately 490,000 datapoints from 160 publications/datasets were gathered and included. The complete dataset is provided in the Supplementary Data 1.

### Emerging contaminant transmission modeling

To estimate the proportion of ECs routed through WWRS, transmission pathways of three representative EC classes—MPs, PhACs, and ARGs—were modeled (Section S1). Each model was parameterized using literature-derived ranges and probability distributions for key transport, transformation, and partitioning processes. Uncertain bounds and distribution for each parameter were defined using standardized criteria to ensure methodological consistency and transparency^58,59,87–89^. Monte Carlo simulations (100,000 iterations) were performed to propagate input uncertainties to the estimated proportion of ECs captured and routed through WWRS.

### Wastewater residual solids management modeling

This study employed the open-source platforms, QSDsan^90,91^ and BioSTEAM^92,93^, to model 40 potential WWRS management systems, including 25 conventional and 15 thermochemical configurations. These systems represent the general breadth of current and emerging WWRS management strategies across different process intensities and technology readiness levels. WWRS were represented using aggregated, representative configurations derived from available data, rather than explicitly resolving all individual WWRS types, sources, or region-specific variations. A modular design framework was adopted, assembling each pathway from combinations of individual unit operations (Table S10). Each unit operation was characterized through mass and energy balances, construction materials, chemical inputs, and product and emission generation. The models capture the full life cycle boundaries of WWRS treatment—from solids collection and preprocessing to end use or disposal—ensuring consistency in resource and emission accounting across all systems. All unit process designs, key assumptions, and parameterization details are documented in Section S2.

### Global WRRF database

The modeled WWRS management systems were applied to water resource recovery facilities (WRRFs) in the HydroWASTE database^94,95^ to assess global transition potential. Residual solids generation for each facility was estimated by multiplying reported influent flow by an empirical yield of 0.907 tonne (1 ton) dry solids per 3785 m^3^ (1 million gallons) of wastewater^96^. Facilities generating less than 5 tonnes dry solids per day were excluded, as these small-scale facilities are generally unsuitable for capital-intensive thermochemical systems^59^. After screening, 4727 WRRFs remained for subsequent analyses. Collectively, these selected facilities represent around 73% of the WWRS flow in the HydroWASTE database, which is considered as sufficient for a first-order global assessment of techno-economic and environmental performance.

### Techno-economic analysis (TEA) and life cycle assessment (LCA)

For each WRRF, 25 conventional and 15 thermochemical systems were simulated to quantify the possible range of costs and carbon intensity (CI) for both approaches. Facility-level results were subsequently aggregated using WWRS mass-flow-weighted averages to generate representative country-level values.

TEA was performed from the WRRF management perspective, focusing only on costs and revenues associated with units owned or operated by WRRFs (e.g., centrifuges, anaerobic digesters, hydrothermal liquefaction reactors). Outsourced processes such as landfilling and land application were included solely for example, tipping fees or transport costs. Country-specific electricity prices were obtained from the World Bank Group^97^. Labor costs were based on U.S. data adjusted by country-average income level from Worlddata^98^; this dataset was selected because it covers more countries with recent-year data, and comparison with the International Labour Organization dataset (ISIC-Rev.4: E. Water supply; sewerage, waste management and remediation activities)^99^ showed reasonable agreement (Pearson’s r = 0.91; Spearman’s ρ = 0.89) in both absolute values and relative rankings (Supplementary Data 1). Construction and chemical costs were also based on U.S. data and adjusted using the country-level price level index (PLI; the ratio between purchasing power parity and currency exchange rate)^100^. This approach provides a transparent and systematic basis for comparing conventional and thermochemical WWRS management systems, while acknowledging that actual labor, equipment, and chemical prices may differ from index-adjusted values. A cash-flow rate-of-return method was used to determine the WWRS management cost ($·tonne^−1^, on a dry-weight basis). All systems were assumed to have a 30-year lifetime, with individual unit lifetimes specified in Table S6. All costs were normalized to 2023 U.S. dollars using the Gross Domestic Product: Chain-type Price Index^101^, the Chemical Engineering Plant Cost Index^102^, and the Employment Cost Index^103^. A complete list of assumptions for TEA can be found in Section S3.

LCA was performed following ISO 14040/14044 guidelines^104,105^ and adopted a full life cycle perspective, encompassing upstream and downstream processes, treatment operations, and credits from displacing products (e.g., electricity, fertilizer). The functional unit was defined as managing 1 dry tonne of WWRS, and the environmental impact indicator was CI (kg CO_2_e·tonne^−1^, on a dry-weight basis). Emission factors for fugitive emissions (e.g., CH_4_ from landfills) were adopted from the BEAM*2024 model^106^. Life cycle inventory data for chemical and energy uses were primarily obtained from the Ecoinvent v3.11 database^107^ and the IPCC 2021 GWP100 (excluding biogenic CO_2_) was used for life cycle impact assessment with regional resolution. Construction-phase emissions were excluded from LCA to maintain consistency with previous studies^58,59^. Detailed LCA information—including data sources, categories, and assumptions—is included in Table S17.

## Supplementary information

Supplementary Information

Description of Additional Supplementary File

Supplementary Data 1

Transparent Peer Review file

## Data Availability

All data supporting the findings of this study are available in the Supplementary Information and the Supplementary Data 1. Source data are provided with this paper.

## Code availability

All code supporting this study is available on GitHub at https://github.com/QSD-Group/EXPOsan/tree/pfas/exposan/htl and archived on Zenodo^108^.
