# Supplementary material for: Thermochemical Treatment of Wastewater Residual Solids for Global Mitigation of Emerging Contaminants
Source: Nat Commun. 2026 Jun 12;17:7462. doi: 10.1038/s41467-026-74242-2 (PMC13408663; doi:10.1038/s41467-026-74242-2)
Supplement: Supplementary file 1 — Supplementary Information [file 41467_2026_74242_MOESM1_ESM.pdf]

Supplementary Information

for

Thermochemical Treatment of Wastewater Residual Solids for Global Mitigation of Emerging Contaminants

Jianan Feng[1,*] and Jeremy S. Guest[1,2,*]

[1] Department of Civil and Environmental Engineering, The Grainger College of Engineering, University of Illinois Urbana-Champaign, Urbana, Illinois 61801, United States

[2] Institute for Sustainability, Energy, and Environment, University of Illinois Urbana-Champaign, Urbana, Illinois 61801, United States

* Corresponding authors: jiananf2@illinois.edu (JF); jsguest@illinois.edu (JSG)

This Supporting Information contains 30 pages, 17 tables, and 3 figures.

S1. Emerging contaminants (ECs) transmission

S1.1. Uncertainty analysis

Given the substantial uncertainties associated with the global ECs transmission, Monte Carlo simulations were employed to estimate of the fraction of ECs routed through wastewater residual solids (WWRS). Parameter ranges and probability distributions were selected following a set of standardized criteria modified from pervious works[1–5]. The following subsections describe the global transmission pathways of three representative EC classes and the derivation of baseline values of each parameter in the transmission models.

Criterion 1. For parameters with published probability distributions, adopt the same distributions as reported in the literature.

Criterion 2. For parameters with only reported ranges, assign a uniform distribution with the minimum and maximum limits as the lower and upper bounds, and their mean as the baseline.

Criterion 3. For parameters with a single empirical value reported, use that value as the baseline and define a uniform distribution spanning 90% to 110% of the baseline.

Criterion 4. For parameters lacking any defined distribution in the literature:

- Criterion 4.1. If more than 20 data points are available, use a triangular distribution with the 5th, 50th, and 95th percentiles of reported values (weighted if needed) as the minimum, most probable, and maximum values.
- Criterion 4.2. If 5 to 20 data points are available, use a triangular distribution with the minimum, mean (weighted if needed), and maximum of reported values as the minimum, most probable, and maximum values.
- Criterion 4.3. If less than 5 data points are available, use a uniform distribution with 80%, 100%, and 120% of the mean value (weighted if needed) as the lower bound, baseline, and the upper bound.

S1.2. Microplastics (MPs)

MPs are emitted from six major sources—textiles, vehicle tires, city dust, road markings, personal care products, and marine coatings (**Table S1**). While secondary MPs derived from macroplastics degradation also contribute to the global burden, their quantification remains unclear; therefore, this analysis focuses primary MPs, for which emission and fate are better characterized[6]. Mass flow rates from each MPs source were compiled from a United Nations report[7]. Among these sources, marine coatings were excluded from water resource recovery facilities (WRRF)-related pathways. MPs from textiles and personal care products were assumed to enter sewer systems at varying regional ratio, with the highest proportion in Western Europe (92%) and the lowest in Africa (3%), from which global mass-flow-weighted averages were calculated as baseline values. For MPs from vehicle tires and road markings, 90% of emissions were assumed to be transported via stormwater and the remainder via wind. Of the stormwater-associated MPs, 68.5% of tire-derived and 23.5% of road-marking-derived MPs were assumed to enter urban sewers, based on the ratio of urban to rural vehicle miles traveled and load lengths in the U.S., respectively. Notably, through U.S. data were used as the baseline, these values were adjusted in the uncertainty analysis to account for the variability among countries. MPs from city dust was assumed to enter

sewers proportionally to impervious surface area, yield a global baseline of 57%. Among all MPs entering sewers, 31.7% (population-weighted average of countries with available data; **Table S2**) were assumed to reach WRRFs via combined sewer systems. Within WRRFs, 95.2% of influent MPs were assumed to partition into WWRS.

Table S1. Parameters of the microplastics (MPs) transmission model.

parameters	unit	crit.	dist.	lower	baseline	upper	ref.
MPs (textiles)	tonne·year^{-1}	#4.3	uniform	398,000	497,000	597,000	[7]
MPs (vehicle tires)	tonne·year^{-1}	#4.3	uniform	1,130,000	1,410,000	1,690,000	[7]
MPs (city dust)	tonne·year^{-1}	#4.3	uniform	520,000	650,000	780,000	[7]
MPs (road markings)	tonne·year^{-1}	#4.3	uniform	472,000	590,000	708,000	[7]
MPs (personal care products)	tonne·year^{-1}	#4.3	uniform	20,000	25,000	30,000	[7]
MPs (marine coatings)	tonne·year^{-1}	#4.3	uniform	40,000	50,000	60,000	[7]
MPs (textiles) to WRRFs	-	#4.3	uniform	0.414	0.517	0.620	[7]
MPs (vehicle tires) to run-off	-	#4.3	uniform	0.72	0.9	1	[6,8]
MPs (vehicle tires) in run-off to sewer	-	#4.3	uniform	0.548	0.685	0.822	[9]
MPs (city dust) to sewer	-	#4.3	uniform	0.456	0.57	0.684	[10]
MPs (road markings) to run-off	-	#4.3	uniform	0.72	0.9	1	[6,8]
MPs (road markings) in run-off to sewer	-	#4.3	uniform	0.188	0.235	0.282	[9]
combined sewer ratio	-	#4.3	uniform	0.254	0.317	0.380	[11–13]
MPs (personal care products) to WRRFs	-	#4.3	uniform	0.398	0.497	0.596	[7]
MPs (marine coatings) to WRRFs	-	-	-	0	0	0	[6]
MPs captured by WWRS in WRRFs	-	#4.1	triangular	0.806	0.952	0.998	[7,14,15]

Table S2. Combined sewer ratio and population in global countries.

country	ratio	population in 2024 (×10^6)	ref.
USA	0.137	340.1	[16]
France	0.7	68.52	[17]
Germany	0.7	83.51	[17]
UK	0.7	69.23	[17]
Denmark	0.45	5.977	[17]
Italy	1	58.99	[17]
China	0.21	1,409	[17]
Ireland	1	5.38	[17]
Japan	0.288	124	[12]
South Korea	0.28	51.75	[18]

S1.3. Pharmaceuticals (PhACs)

PhACs enter the environment as unused products or as excreted compounds (**Table S3**). Unused PhACs follow three disposal pathways: take-back to pharmacies, disposal in trash, or flushing into toilets, with the global population-weighted average ratios of 16.9% for the take-back pathway and 39.9% for the ratio between toilet and trash pathways (**Table S4**). Take-back PhACs were assumed to undergo complete destruction through incineration. Trashed PhACs were either incinerated or landfilled, with negligible direct release as illustrated in Cook *et al.*[19] Around half of

the flushed PhACs were assumed to reach WRRFs (consistent with the global collection ratio for MPs from personal care products; **Table S1**), with the remainder discharged to the environment. On the other hand, used PhACs were divided into human- and animal-derived fractions according to their global market shares (98%[20] and 2%[21], respectively). For human consumption, 39% of used PhACs were assumed to be excreted, with the excreted PhACs reaching WRRFs at the same collection ratio as flushed unused PhACs. PhACs consumed by animals were assumed to be excreted at the same ratio as humans, with all excreta released into the environment. Within WRRFs, 78.4% of PhACs were removed through adsorption to WWRS and biotransformation (i.e., oxidation or assimilation into biomass), with an adsorption-to-biotransformation ratio of 2.91.

Table S3. Parameters of the pharmaceuticals (PhACs) transmission model.

parameters	unit	crit.	dist.	lower	baseline	upper	ref.
unused PhACs ratio	-	#2	uniform	0.15	0.565	0.98	[22]
unused PhACs take-back ratio	-	#4.3	uniform	0.136	0.169	0.203	[23–30]
unused PhACs toilet-to-trash ratio	-	#4.3	uniform	0.319	0.399	0.479	[23–30]
unused PhACs toilet to WRRFs	-	#4.3	uniform	0.398	0.497	0.596	[7]
used PhACs human	-	#4.3	uniform	0.784	0.98	1	[20,21]
used PhACs human excretion	-	#4.3	triangular	0.02	0.39	0.862	[31]
used PhACs human excretion to WRRFs	-	#4.3	uniform	0.398	0.497	0.596	[7]
used PhACs animal excretion	-	#4.3	triangular	0.02	0.39	0.862	[31]
used PhACs animal excretion to WRRFs	-	-	-	0	0	0	-
PhACs WRRF removal ratio	-	#4.2	triangular	0.15	0.784	1	[19]
PhACs WRRF adsorption-biotransformation ratio	-	#4.2	triangular	0.168	2.91	7	[19]

Table S4. Disposal pathways for unused pharmaceuticals and population in global countries.

country	take-back ratio	toilet-trash ratio	population in 2024 ($\times10^6$)	ref.
UK	0.27	0.55	69.23	[32–35]
Brazil	0.25	0.11	212	[36]
India	0.16	0.39	1,451	[37]
Australia	0.34	0.25	27.2	[33]
South Korea	0.28	0.03	51.75	[33]
Sweden	0.88	0	10.57	[33,35]
Portugal	0.69	0	10.7	[33]
Netherlands	0.81	0.30	17.99	[33,35]
New Zealand	0.20	0.44	5.338	[33,35]
USA	0.13	0.72	340.1	[33,35]
Poland	0.08	0.51	36.55	[33]
Ireland	0.06	0.84	5.38	[33]
Israel	0.07	0.05	9.974	[33]
Latvia	0.10	0.29	1.862	[33]
Lithuania	0.01	0.06	2.888	[33,35]
Ethiopia	0	0.26	132.1	[38]
Kuwait	0.06	0.08	4.974	[35]
Liberia	0.10	0.42	5.613	[39]

S1.4. Antibiotic resistance genes (ARGs)

Animal and human wastes are the two primary sources of ARGs[40]. Due to the scarcity of global ARGs transmission data, antibiotics were used as proxies given their strong linkage with resistomes (**Table S5**)[41]. Globally, approximately 72% of antibiotic consumption is attributed to livestock[42], making animal sources the dominant contributor as antibiotics originating from animals were assumed to be released directly to the environment. On the other hand, antibiotics from human excreta were modeled identically to general PhACs, while unused antibiotics were excluded because their contributions to ARG proliferation remain unclear. Within WRRFs, 90% of influent ARGs were assumed to be captured by WWRS, consistent with literature-reported removal efficiencies. The remaining fraction was considered to be discharged with the effluent, under the conservative assumption no ARGs degradation occurs during wastewater treatment.

Table S5. Parameters of the antibiotic resistance genes (ARGs) transmission model.

parameters	unit	crit.	dist.	lower	baseline	upper	ref.
antibiotic human consumption ratio	-	#4.3	uniform	0.224	0.28	0.336	42
used antibiotics human excretion	-	#4.3	triangular	0.02	0.39	0.862	31
used antibiotics human excretion to WRRFs	-	#4.3	uniform	0.398	0.497	0.596	7
used antibiotics animal excretion	-	#4.3	triangular	0.02	0.39	0.862	31
used antibiotics animal excretion to WRRFs	-	-	-	0	0	0	-
ARGs captured by WWRS in WRRFs	-	#2	uniform	0.925	0.90	0.95	43

S1.5. Per- and polyfluoroalkyl substances (PFAS)

Two of the most widely recognized PFAS compounds—perfluorooctanoic acid (PFOA) and perfluorooctane sulfonate (PFOS)—were examined trace their release pathways to the environment. In contrast to MPs, PhACs, and ARGs, which are strongly associated with municipal wastewater streams, PFOA and PFOS originate predominantly from industrial activities. For instance, Liu *et al.* reported over 85% of PFOA and PFOS in the Chinese environment stem from industrial discharges, whereas contributions routed through WWRS account for only about 0.1%[44]. However, this estimate does not capture the potential influence of aqueous film-forming foams (AFFF), which can enter sewer systems via runoffs. In scenarios where industrial emissions are effectively phased out—such as through the production and use bans already implemented in U.S. and parts of Europe[45]—the relative importance of WWRS in capturing residual PFAS becomes more pronounced. When assuming 50% of AFFF-related runoff enters WRRFs, the proportion of PFOA and PFOS intercepted within WWRS could increase to approximately 0.6% and 3.8%, respectively. While these fractions remain modest compared to other ECs, they underscore the complementary role of WWRS as a secondary barrier within a broader, multi-sectoral framework for mitigating legacy and diffuse PFAS emissions.

S2. Unit processes and system configurations

S2.1. General statement

The modeling of unit processes (**Table S6**) and systems (**Table S10**) was conducted using QSDsan[46,47] and BioSTEAM[48,49], with all relevant code available on GitHub at https://github.com/QSD-Group/EXPOsan/tree/pfas/exposan/htl. Detailed descriptions of each unit process are presented in the following subsections.

Table S6. Modeling assumptions and references of each unit process in wastewater residual solids management. Abbreviations: anaerobic digestion (AD), hydrothermal liquefaction (HTL), hydrothermal alkaline treatment (HALT), catalytic hydrothermal gasification (CHG), supercritical water oxidation (SCWO), combined heat and power (CHP), Biosolids Emissions Assessment Model version 2024 (BEAM*2024), ruthenium on a carbon support (Ru/C).

unit process	lifetime [year]	resource recovery	greenhouse gas emission			ref.	
			scope 1	scope 2[a]	scope 3	capital cost	GHG emission
thickening	25[50]	-	-	electricity	polyacrylamide	Seider *et al.*, 2017[51]	BEAM*2024[52,53]
aerobic digestion	30	-	-	electricity	-	Shoener *et al.*, 2016[54]	BEAM*2024[52,53]
AD	30[55]	energy	CH_4	electricity	-	Shoener *et al.*, 2016[54]	BEAM*2024[52,53]
dewatering	20[56]	-	CH_4	electricity	polyacrylamide	Seider *et al.*, 2017[51]	BEAM*2024[52,53]
lime stabilization	25	-	-	electricity	quicklime	Williford *et al.*, 2007[57]	BEAM*2024[52,53]
composting	25	nutrient	CO_2, CH_4, N_2O	-	diesel, sawdust	Stramer *et al.*, 2010[58]	BEAM*2024[52,53]
heat drying	25	-	CO_2	electricity, natural gas	-	Hao *et al.*, 2020[59]	BEAM*2024[52,53]
landfilling	-	energy	CH_4, N_2O	-	-	-	BEAM*2024[52,53]
land application	-	nutrient	CO_2, CH_4, N_2O	-	diesel	-	BEAM*2024[52,53]
incineration	20[60]	energy	CO_2, CH_4, N_2O	electricity, NG	-	Gergel, 2015[61]	BEAM*2024[52,53]
HTL	20	energy	-	electricity	-	Jones *et al.*, 2014[62]	this work
HALT	15	energy	-	electricity	NaOH, HCl	Jones *et al.*, 2014[62]	this work
CHG	20	energy	-	electricity	7.8% Ru/C	Jones *et al.*, 2014[62]	this work
SCWO	15	-	-	electricity	-	Qiu *et al.*, 2024[63]	this work
pyrolysis	20	energy	-	electricity	-	Khan *et al.*, 2022[64]	this work, BEAM*2024[52,53]
gasification	20	energy	-	electricity	-	Clack *et al.*, 2024[65]	Swanson *et al.*, 2010[66], BEAM*2024[52,53]
CHP	20[67]	-	CO_2	natural gas	-	Shoener *et al.*, 2016[54]	this work
cooling tower	20[68]	-	-	electricity	water	Humbird *et al.*, 2011[69]	this work
transportation	-	-	-	-	transportation	-	Ecoinvent[70]

[a] Heating and cooling demands are only considered for individual unit process in systems without a CHP unit or cooling tower and are excluded from the table.

S2.2. Thickening

The influent to the thickener is raw sludge. Both centrifugal and non-centrifugal thickeners (including gravity, gravity belt, and rotary drum types) were modeled; however, non-centrifugal thickeners were selected as the default configuration due to their substantially lower energy intensity (4.9 $kW \cdot tonne^{-1}$ sludge; reported on a dry-weight basis unless otherwise noted) compared with centrifugal units (33 $kW \cdot tonne^{-1}$ sludge). A polymer (polyacrylamide) dosage of 5 $kg \cdot tonne^{-1}$ sludge was applied for conditioning. The max hydraulic loading of each thickening unit was set at 100 $m^3 \cdot h^{-1}$, and the thickened sludge was assumed to reach a moisture content of 97%. Reject water from thickening was assumed to be recycled to the WRRF headworks.

S2.3. Aerobic digestion

The influent to the aerobic digester is thickened sludge. During aerobic digestion, 47.5% of the volatile solids (VS, equivalent to ash-free dry solids) were assumed degraded. The produced gas is primarily biogenic CO_2, which is not considered a GHG emission in this study. The digested solids are thereby referred to as biosolids. Aeration is supplied by blowers, which requires an electricity input of 0.03 $kW \cdot m^{-3}$ reactor volume. The reactor volume was calculated based on the influent flow rate and a hydraulic retention time (HRT) of 14 days[71]. The reactor has an assumed depth of 10 m, with structural specifications including a 0.15 m concrete wall thickness, a 0.23 m concrete slab thickness, and a 0.91 m freeboard above the liquid level. The solids retention time (SRT) was set at 40 days to ensure sufficient biomass degradation. There is no heating requirement for aerobic digestion, and water loss during the process was assumed negligible.

S2.4. Anaerobic digestion

The influent to the anaerobic digester is thickened sludge. During anaerobic digestion, 42.5% of the VS (equivalent to ash-free dry solids) were assumed degraded. The produced biogas consists primarily of CH_4 and biogenic CO_2, with CH_4 accounting for 65% of the gas volume. Biogas utilization was assumed as follows: (1) 1% fugitive emissions, (2) 9% flared onsite, and (3) 90% either combusted in a combined heat and power (CHP) unit (if available) or upgraded and injected into renewable natural gas (RNG) pipelines. A 1% fugitive loss was applied to flared CH_4. For RNG pathways, a parasitic loss of 16.9% was assumed for gas conditioning and pipeline injection. The digested solids after anaerobic digestion are referred to as biosolids. Mixing is provided by vertical mixers, with an electricity requirement of 0.0065 $kW \cdot m^{-3}$ reactor volume. The reactor volume was determined based on the influent flow rate and a HRT of 22 days, which equals the SRT[71]. The reactor depth was assumed to be 10 m, with structural parameters including a 0.15 m concrete wall thickness, a 0.23 m concrete slab thickness, and a 0.91 m freeboard above the liquid surface. The reactor operates at a mesophilic temperature of 35 °C. As with aerobic digestion, water loss during anaerobic digestion was assumed negligible.

S2.5. Dewatering

The influent to the dewatering unit is either thickened sludge or biosolids. For anaerobically digested biosolids, 90% of the entrapped CH_4 (at a concentration of 6 g $CH_4 \cdot m^{-3}$ biosolids) was assumed to be released during dewatering. Polyacrylamide polymer is applied for conditioning at a dosage of 5 $kg \cdot tonne^{-1}$ sludge. The electricity demand was assumed to be 1.4 $kWh \cdot m^{-3}$ sludge.

The resulting dewatered solids were assumed to have a moisture content of 80%. Reject water from dewatering was assumed to be recycled to the WRRF headworks.

S2.6. Lime stabilization

Lime stabilization, composting (**Section S2.7**), and heat drying (**Section S2.8**) are mutually exclusive processes in conventional wastewater residual solids treatment, as all serve as final stabilization steps with overlapping objectives including pathogen reduction, odor control, and preparation for end use. Additionally, lime stabilization is typically not applied in conjugation with digestion due to redundant stabilization functions. The influent to the lime stabilization unit is therefore dewatered sludge. Quicklime dosage was set at 0.3 $tonne \cdot tonne^{-1}$ and electricity requirements was assumed to be 3.7 $kWh \cdot wet\ tonne^{-1}$ sludge. The lime stabilized solids are thereby referred to as biosolids.

S2.7. Composting

The influent to the composting unit is dewatered sludge or biosolids. A bulking agent (e.g., sawdust) with a moisture content of 39% and a C:N mass ratio above 200 is added at a volumetric ratio of 3 $m^3 \cdot m^{-3}$ dewatered solids to improve porosity and moisture balance. The bulking agent was assumed to be ground onsite with a diesel requirement of 3.3 $L \cdot wet\ tonne^{-1}$ bulking agent. Additional diesel requirements depend on the composting configuration: 5 $L \cdot wet\ tonne^{-1}$ blended feedstock for windrow composting, and 2.5 $L \cdot wet\ tonne^{-1}$ blended feedstock for aerated static pile (ASP) composting and 2 in-vessel composting. Electricity requirements also vary by system, with 0 $kWh \cdot tonne^{-1}$ solids (excluding the bulking agent) for windrow composting, 180 $kWh \cdot tonne^{-1}$ solids for ASP composting, and 291 $kWh \cdot tonne^{-1}$ solids for in-vessel composting. Windrow composting was selected as the default configuration due to its simplicity and low energy intensity. Assuming well-aerated conditions, the emission factor for fugitive CH_4 was 0.0001 g $CH_4 \cdot g^{-1}$ C. For digested input solids, the fugitive N_2O emission factors was 0.00076 g $N_2O \cdot g^{-1}$ N, while for undigested solids, it was 0.018 g $N_2O \cdot g^{-1}$ N. The produced compost was assumed to be transported for land application (**Section S1.10**).

S2.8. Heat drying

The influent to the heat drying unit is dewatered sludge or biosolids. The input solids are dried to a target moisture content of 20% at an operating temperature of 90 °C, requiring 4.5 $GJ \cdot tonne^{-1}$ water removed for heating and 214 $kWh \cdot tonne^{-1}$ solids for electricity. The vaporized water was assumed to be released to the atmosphere.

S2.9. Landfilling

The influent to the landfill is dewatered sludge or biosolids. The extent of organic carbon decomposition varies with feedstock type, with 50% assumed for digested biosolids and 80% for undigested sludge. As a result of organic carbon decomposition, landfill gas (LFG) is generated, which consists of 50% CH_4 by weight. LFG production was modeled as a time-dependent process following the 'typical' scenario in the U.S. EPA Waste Reduction Model (**Table S7**)[72]. A first-order decay rate constant of 0.18 $year^{-1}$ and a methane correction factor of 1.0 were applied. Collected LFG was assumed to be combusted, with a 1% of fugitive lost rate. The remaining LFG was converted to electricity at an energy conversion efficiency of 0.0000854 $kWh \cdot BTU^{-1}$ and a net

capacity factor of 0.85. When the C:N mass ratio of the landfilled solids was below 30, fugitive N_2O emissions were estimated using an emission factor is 0.015 g N_2O-N·g^{-1} N; otherwise, N_2O generation was neglected. The carbon sequestration potential was quantified as the CO_2-equivalent of the residual organic carbon stably retained within the landfill matrix.

Table S7. Landfill gas generation and lost schedules.

years after landfilling	captured	lost	CH_4 oxidized
0-1	0%	100%	10%
2-4	50%	50%	25%
5-14	75%	25%	25%
after capping	90%	10%	35%

S2.10. Land application

The influent to the land application is dewatered biosolids, which were assumed to be stored for 10 days prior to land application. When the solids content of biosolids is below 55%, fugitive CH_4 and N_2O emissions occurs during storage, with emission factors of 0.0091 kg CH_4·m^{-3}·day^{-1} and 0.00043 kg N_2O·m^{-3}·day^{-1}, respectively; otherwise, there emissions were neglected. During land application, when the C:N mass ratio of biosolids is below 30, fugitive N_2O emission was estimated using an emission factor of 0.0275 g N_2O-N·g^{-1} N for fine-textured soil (assumed representing 50% of all soils) and 0 g N_2O-N·g^{-1} N for coarse-textured soils. The fugitive N_2O emission was further adjusted at a reduction ratio (*R*) as a function of the solids content (*S*) of biosolids (**Eq. S1**). The diesel requirement for biosolids spreading was estimated based on the biosolids volumetric flow rate, tractor load size (13 m^3·$load^{-1}$), load frequency (3 load·h^{-1}), and fuel consumption rate (25 L diesel·h^{-1}). Nutrients in land-applied biosolids were assumed to offset the use of synthetic nitrogen and phosphorus fertilizers and the carbon sequestration potential was estimated as 0.4475 tonne CO_2e·$tonne^{-1}$ biosolids.

$$R = \begin{cases} 1 & (S \geq 0.8) \\ 0.276S - 0.1518 & (0.8 > S \geq 0.55) \\ 0 & (S < 0.55) \end{cases} \qquad \textbf{Eq. S1}$$

S2.11. Incineration

The influent to the incinerator is dried sludge or biosolids after heat drying. Fugitive CH_4 emission during incineration was assumed to be 9.7 g CH_4·$tonne^{-1}$ solids. The emission factor of fugitive N_2O emission (EF_{N_2O}, [g N_2O-N·g^{-1} N]) depends on the incineration temperature (*T*, [K]) and was calculated using **Eq. S2**. The default incineration temperature was set at 850 °C. Additionally, an N_2O emission reduction ratio (*R*) was applied based on the solids content of input solids (*S*), according to **Eq. S3**. The total energy potential of the solids was assumed to be 23 GJ·$tonne^{-1}$ for sludge and 12 GJ·$tonne^{-1}$ for biosolids. During the incineration, all VS were considered fully degraded. It was assumed 50% of the energy produced can be captured, with a conversion efficiency of 80%, to support continuous operation of the incinerator. The heating energy required for incineration was calculated based on water removal (4.5 GJ·$tonne^{-1}$ water). If the recovered energy was insufficient to meet this requirement, additional natural gas was supplied; otherwise, excess heat was released to the atmosphere.

$$EF_{N_2O} = \max(0, 1.613 - 0.0014 \times (\max(T, 750) + 273.15)) \quad \textbf{Eq. S2}$$

$$R = \begin{cases} 0.6 & (S \geq 0.87) \\ 0.5 & (0.87 > S \geq 0.24) \\ 0 & (S < 0.24) \end{cases} \quad \textbf{Eq. S3}$$

S2.12. Hydrothermal liquefaction (HTL)

The influent to the HTL unit is dewatered sludge or biosolids originating from aerobic or anaerobic digestion. Conversion of solids into biocrude, an aqueous phase, off-gas, and hydrochar was assumed to occur within 15 min at 350°C under autogenous pressure. Product yields were estimated using a multiphase component additivity (MCA) model (**Table S8**), with the biochemical composition of solids as model inputs[73–75]. The biochemical composition of sludge was assumed to comprise 23.1% ash (dry-weight basis), 20.6% lipid, 45.6% protein, and 33.8% carbohydrate (all on an ash-free dry-weight basis). The biochemical composition of sludge after digestion (biosolids) was adjusted based on the VS reduction. The biocrude, with a higher heating value (HHV) of 44.5 MJ·kg^{-1} and a density of 983 kg·m^{-3}, was assumed to be transported to petroleum refineries for upgrading; the hydrochar was landfilled; the aqueous was sent to a catalytic hydrothermal gasification (CHG) unit (**Section S2.13**); and the off-gas, with the composition listed in **Table S9**, was assumed to be sent to a combined heat and power (CHP) unit (**Section S18**).

Table S8. Parameters of the multiphase component additivity model.

product	HTL			HALT factor
	lipid	protein	carbohydrate	
biocrude	0.846	0.445	0.205	0.85
aqueous	0.154	0.481	-	-
off-gas	-	0.074	0.418	1
hydrochar	-	-	0.377	0.2

Table S9. Compositions of gas products from thermochemical units. Abbreviations: hydrothermal liquefaction (HTL), hydrothermal alkaline treatment (HALT), catalytic hydrothermal gasification (CHG), supercritical water oxidation (SCWO), mass ratio (M), volume ratio (V).

gas	HTL[62]		HALT[76]		CHG[62]		SCWO[77]		pyrolysis[77]		gasification[78]	
	M	V	M	V	M	V	M	V	M	V	M	V
CH_4	0.05	0.125	0	0	0.527	0.751	0	0	0.073	0.119	0.02	0.025
CO	0	0	0	0	0	0	0	0	0.238	0.222	0.58	0.413
CO_2	0.918	0.833	0	0	0.432	0.224	1	1	0.549	0.326	0.36	0.163
C_2H_6	0.032	0.043	0	0	0.011	0.008	0	0	0.049	0.043	0	0
C_3H_8	0	0	0	0	0.030	0.016	0	0	0.072	0.043	0	0
H_2	0	0	1	1	0.000	0.001	0	0	0.019	0.248	0.04	0.399

S2.13. Hydrothermal alkaline treatment (HALT)

HALT operates under conditions similar to HTL, but with the addition of 2 M NaOH as a catalytic base and 2 M HCl added for subsequent neutralization. These chemical additions alter product yields, and the product distribution estimated by the MCA model was adjusted using an empirical

'HALT factor', while the aqueous-phase yield was determined by mass balance (**Table S8**). Unlike HTL, the hydrochar produced from HALT was assumed to be suitable for land application due to the expected more complete removal of ECs[79]. The composition of produced gas was also updated to reflect the altered reaction chemistry (**Table S9**).

S2.14. Catalytic hydrothermal gasification (CHG)

The influent to the CHG unit is the aqueous product from HTL, characterized by a high chemical oxygen demand (COD). CHG is employed to recover energy value by mineralizing and gasifying the residual dissolved organics (typically in the $g \cdot L^{-1}$ range) into fuel gases with a 7.8% Ru/C catalyst (7.8% Ruthenium on a carbon support). The produced gas-liquid mixture was passed through a flash vessel for vapor-liquid separation and the produced fuel gases—assumed to have the composition listed in **Table S9**—were sent to a CHP unit (**Section S2.18**). The liquid effluent from the flash vessel was assumed to be recycled to the WRRF headworks.

S2.15. Supercritical water oxidation (SCWO)

The influent to the SCWO unit is dewatered sludge or biosolids originating from aerobic or anaerobic digestion. The reaction was operated at 550 °C under autogenous pressure. All VS were assumed to be fully mineralized to CO_2 and H_2O, while the inorganic fraction was retained as ash and subsequently landfilled.

S2.16. Pyrolysis

The influent to the pyrolysis unit is dried sludge or biosolids originating from aerobic or anaerobic digestion after heat drying. The reaction temperature was set at 600 °C. At this temperature, product yields were assumed to be 0.43 tonne $biochar \cdot tonne^{-1}$ solids, 0.37 tonne $bio\text{-}oil \cdot tonne^{-1}$ solids, and 0.2 tonne $pyrogas \cdot tonne^{-1}$ solids[77]. The biochar was assumed to be land-applied; the bio-oil, with a HHV of 33.7 $MJ \cdot kg^{-1}$ and a density of 1,070 $kg \cdot m^{-3}$ [77], was assumed to be sent to petroleum refineries for upgrading; and the pyrogas (composition summarized in **Table S9**) was directed to a CHP unit (**Section S2.18**). The electricity requirement for pyrolysis was estimated at 123 $kWh \cdot tonne^{-1}$ solids[53].

S2.17. Gasification

The influent to the gasification unit is dried sludge or biosolids originating from aerobic or anaerobic digestion after heat drying. The reaction temperature was set at 900 °C. At this temperature, the tar yield was assumed to be 0.019 $tonne \cdot tonne^{-1}$ VS, with the remaining VS converted to syngas and the inorganic fraction retained as ash[80]. The tar was assumed to be treated and disposed similarly to coal tar; the ash was landfilled; and the syngas (composition summarized in **Table S9**) was directed to a CHP unit (**Section S2.18**). The electricity requirement for gasification was estimated at 302 $kWh \cdot tonne^{-1}$ solids[66].

S2.18. Combined heat and power (CHP)

Fuel gases produced from anaerobic digestion (if not directed to RNG production), HTL, HALT, CHG, pyrolysis, and gasification were collected and combusted in a CHP unit to recover thermal and electricity energy. The primary function of the CHP unit was to meet onsite heat demands of the corresponding processes. When the energy content of the feed gases was insufficient,

supplemental natural gas was assumed to be purchased to close the heating deficit. Conversely, when excess heat was available, additional electricity was generated. However, the CHP unit was not assumed to purchase natural gas solely for electricity production; instead, any additional electricity demand was met through direct purchase from the grid.

S2.19. Transportation

Transportation was assumed be carried out by trucks. Since WWRF-specific transportation distances were difficult to obtain and transportation has been shown to contribute negligibly to both costs and GHG emissions in WWRS management[3], a default distance of 100 km was assumed for all products and wastes.

S2.20. System configurations

The unit processes described in this section were systematically combined to generate 25 conventional and 15 thermochemical WWRS management systems (**Table S10**), encompassing a broad range of treatment and resource recovery configurations.

Table S10. Conventional and thermochemical wastewater residual solids management pathways. Solid and blank circles denote the presence and absence of corresponding units, respectively. Only major process units are shown. Abbreviations: combined heat and power (CHP), hydrothermal liquefaction (HTL), catalytic hydrothermal gasification (CHG), hydrothermal alkaline treatment (HALT), supercritical water oxidation (SCWO).

code	thickening	digestion	dewatering	lime stabilization	composting	heat drying	valorization or disposal	CHP
conventional pathways (C)								
C1	●	○	●	○	○	○	landfilling	○
C2	●	○	●	●	○	○	landfilling	○
C3	●	○	●	●	○	○	land application	○
C4	●	○	●	○	●	○	land application	○
C5	●	○	●	○	○	●	landfilling	○
C6	●	○	●	○	○	●	land application	○
C7	●	○	●	○	○	●	incineration	○
C8	●	aerobic	●	○	○	○	landfilling	○
C9	●	aerobic	●	○	○	○	land application	○
C10	●	aerobic	●	○	●	○	land application	○
C11	●	aerobic	●	○	○	●	landfilling	○
C12	●	aerobic	●	○	○	●	land application	○
C13	●	aerobic	●	○	○	●	incineration	○
C14	●	anaerobic	●	○	○	○	landfilling	○
C15	●	anaerobic	●	○	○	○	land application	○
C16	●	anaerobic	●	○	●	○	land application	○
C17	●	anaerobic	●	○	○	●	landfilling	○
C18	●	anaerobic	●	○	○	●	land application	○
C19	●	anaerobic	●	○	○	●	incineration	○
C20	●	anaerobic	●	○	○	○	landfilling	●
C21	●	anaerobic	●	○	○	○	land application	●
C22	●	anaerobic	●	○	●	○	land application	●

C23	●	anaerobic	●	○	○	●	landfilling	●
C24	●	anaerobic	●	○	○	●	land application	●
C25	●	anaerobic	●	○	○	●	incineration	●
			thermochemical pathways (T)					
T1	●	○	●	○	○	○	HTL + CHG	●
T2	●	○	●	○	○	○	HALT + CHG	●
T3	●	○	●	○	○	○	SCWO	○
T4	●	○	●	○	○	●	pyrolysis	●
T5	●	○	●	○	○	●	gasification	●
T6	●	aerobic	●	○	○	○	HTL + CHG	●
T7	●	aerobic	●	○	○	○	HALT + CHG	●
T8	●	aerobic	●	○	○	○	SCWO	○
T9	●	aerobic	●	○	○	●	pyrolysis	●
T10	●	aerobic	●	○	○	●	gasification	●
T11	●	anaerobic	●	○	○	○	HTL + CHG	●
T12	●	anaerobic	●	○	○	○	HALT + CHG	●
T13	●	anaerobic	●	○	○	○	SCWO	●
T14	●	anaerobic	●	○	○	●	pyrolysis	●
T15	●	anaerobic	●	○	○	●	gasification	●

S3. Techno-economic analysis (TEA)

S3.1. Cost indices

All costs in this section are referenced to U.S. conditions and expressed in 2023 U.S. dollars. For country-level analysis, U.S. data were adjusted using the most recent price level index and national income level (**Supplementary Data 1**). If no such data was available, regional averages or values from economically similar countries were used.

S3.2. Unit costs

The purchase costs of individual unit processes were compiled from multiple literature sources (**Table S6**). To support future applications involving scalable cost estimation across a wide range of design capacities, the relationship between the purchase cost or installed cost and the scaling stream for each unit process was fitted to a power function, as summarized in **Table S11**. When the relationship between installed cost and the scaling stream is unavailable, a bare module factor (the ratio between installed cost and purchase cost) is provided in the same table.

Table S11. Purchase-cost and installed-cost power functions for unit processes in wastewater residual solids (WWRS) management systems. Power-function parameters may vary slightly depending on the source system from which they were derived. Abbreviations: hydrothermal liquefaction (HTL), catalytic hydrothermal gasification (CHG), hydrothermal alkaline treatment (HALT), supercritical water oxidation (SCWO), combined heat and power (CHP), wastewater residual solids (WWRS), purchase cost (*PC*), installed cost (*IC*), scaling stream magnitude (*Q*).

unit process	scaling stream	unit	equation	bare module factor
thickening	raw WWRS	wet tonne·h^{-1}	$PC = 4{,}430 \times Q^{0.501}$	2.93
aerobic digestion	thickened WWRS	wet tonne·h^{-1}	$PC = 68{,}700 \times Q^{0.662}$	2.01
anaerobic digestion	thickened WWRS	wet tonne·h^{-1}	$PC = 63{,}200 \times Q^{0.828}$	1.95
dewatering	thickened WWRS	wet tonne·h^{-1}	$PC = 19{,}400 \times Q^{0.463}$	2.15
lime stabilization	dewatered WWRS	wet tonne·h^{-1}	$IC = 1{,}350{,}000 \times Q^{0.562}$	-
composting	dewatered WWRS	wet tonne·h^{-1}	$IC = 1{,}680{,}000 \times Q^{0.371}$	-
heat drying	dewatered WWRS	wet tonne·h^{-1}	$PC = 631{,}000 \times Q^{0.7}$	3.17
incineration	dried WWRS	wet tonne·h^{-1}	$PC = 5{,}120{,}000 \times Q^{0.775}$	2
HTL	dewatered WWRS	wet tonne·h^{-1}	$PC = 2{,}040{,}000 \times Q^{0.704}$	1.87
CHG	HTL aqueous	wet tonne·h^{-1}	$PC = 1{,}090{,}000 \times Q^{0.647}$	2.02
HALT	dewatered WWRS	wet tonne·h^{-1}	$PC = 2{,}660{,}000 \times Q^{0.708}$	1.88
SCWO	dewatered WWRS	wet tonne·h^{-1}	$PC = 4{,}740{,}000 \times Q^{0.7}$	2
pyrolysis	dried WWRS	wet tonne·h^{-1}	$PC = 2{,}470{,}000 \times Q^{0.7}$	2
gasification	dried WWRS	wet tonne·h^{-1}	$PC = 5{,}200{,}000 \times Q^{0.7}$	2
CHP	power generated	kW	$IC = 1{,}225 \times Q$	-
cooling tower	makeup water	wet tonne·h^{-1}	$PC = 82{,}800 \times Q^{0.709}$	1.63

S3.3. Chemical and service costs

The chemical and service costs listed in **Table S12** represent typical market prices and serve as the basis for estimating the variable costs associated with each treatment process.

Table S12. Chemical and service cost data. Abbreviations: catalytic hydrothermal gasification (CHG).

item	unit	price	ref.
polyacrylamide	$\$\cdot kg^{-1}$	7.21	Davis *et al.*, 2018[81]
quicklime	$\$\cdot kg^{-1}$	0.326	Davis *et al.*, 2018[81]
sawdust	$\$\cdot kg^{-1}$	0.0405	Kline *et al.*, 2008[82]
CHG catalyst	$\$\cdot kg^{-1}$	177	Jones *et al.*, 2014[62]
NaOH	$\$\cdot kg^{-1}$	0.654	Davis *et al.*, 2018[81]
HCl (pure basis)	$\$\cdot kg^{-1}$	1.25	Davis *et al.*, 2024[83]
crude oil[a]	$\$\cdot barrel^{-1}$	76.1	U.S. EIA[84]
tar management	$\$\cdot kg^{-1}$	0.405	ProfitableVenture[85]
hydrochar[b]	$\$\cdot kg^{-1}$	0.0556	Gu *et al.*, 2020[86]
biochar[b]	$\$\cdot kg^{-1}$	0.131	Cloverly[87]
deionized water	$\$\cdot kg^{-1}$	0.000549	Davis *et al.*, 2018[81]
cooling tower chemicals	$\$\cdot kg^{-1}$	4.90	Davis *et al.*, 2018[81]
landfill disposal	$\$\cdot kg^{-1}$	0.0522	Davis *et al.*, 2018[81]
diesel	$\$\cdot gallon^{-1}$	4.22	U.S. EIA[88]
tipping fee	$\$\cdot$wet kg^{-1}	0.0626	EREF[89]
biosolids	$\$\cdot$wet kg^{-1}	0	-
compost	$\$\cdot$wet kg^{-1}	0.05	-
natural gas	$\$\cdot kmol^{-1}$	3.50	BioSTEAM[90]
electricity	$\$\cdot kWh^{-1}$	**Supplementary Data 1**	
heating/cooling utilities	$\$\cdot kmol^{-1}$	listed in _heat_utility.py in the ref.	BioSTEAM[90]
solids transportation	$\$\cdot kg^{-1}$	0.00551 + 0.0000541 × distance [km]	Marufuzzaman *et al.*, 2015[91]
liquids transportation	$\$\cdot m^{-3}$	7.88 + 0.0973 × distance [km]	Pootakham and Kumar, 2010[92]

[a] Crude oil price was converted to biofuel prices based on their higher heating values.
[b] Hydrochar produced from hydrothermal alkaline treatment (HALT) was assumed to be sold at the price listed, while hydrochar produced from hydrothermal liquefaction (HTL) was assumed to be landfilled.

S3.4. Labor costs

Labor costs for WWRS management systems were differentiated between conventional and thermochemical systems to reflect the additional supervision, operation, and maintenance requirements of the latter. For both system types, labor costs were categorized into managerial staff (plant manager, plant engineer, maintenance supervisor, lab manager) and operational staff (shift supervisor, lab technician, maintenance technician, shift operator, yield employee, clerk and secretary). Managerial staffing levels were assumed constant across plant sizes, while operational staffing scaled linearly with the dry sludge mass flow rate, referenced to a facility treating 100 tonnes of sludge per day[93]. The estimated full-time equivalent (FTE) allocations by position and unit process, along with corresponding labor costs, are included in **Table S13**. All labor costs were adjusted to 2023 U.S. dollars using the labor index retrieved from U.S. Bureau of Labor Statistics[94]. A summary of total labor costs for each system is presented in **Table S14**.

Table S13. Staffing levels and labor costs for wastewater residual solids management unit processes.

<table>
<tr><th rowspan="2">unit process</th><th colspan="4">fixed staffing</th><th colspan="6">variable staffing at 100 tonne sludge·day^{-1}</th></tr>
<tr><th>plant manager</th><th>plant engineer</th><th>maintenance supervisor</th><th>lab manager</th><th>shift supervisor</th><th>lab technician</th><th>maintenance technician</th><th>shift operator</th><th>yield employee</th><th>clerk and secretary</th></tr>
<tr><td colspan="11">full-time equivalent (FTE)</td></tr>
<tr><td>thickening</td><td rowspan="6">0.05</td><td rowspan="6">0.05</td><td rowspan="6">0.05</td><td rowspan="6">0.05</td><td rowspan="6">0.15</td><td rowspan="6">0.05</td><td rowspan="6">0.05</td><td rowspan="6">0.2</td><td rowspan="6">0.05</td><td rowspan="6">0.05</td></tr>
<tr><td>dewatering</td></tr>
<tr><td>lime stabilization</td></tr>
<tr><td>composting</td></tr>
<tr><td>landfilling</td></tr>
<tr><td>land application</td></tr>
<tr><td>aerobic digestion</td><td rowspan="5">0.15</td><td rowspan="5">0.15</td><td rowspan="5">0.15</td><td rowspan="5">0.15</td><td rowspan="5">0.45</td><td rowspan="5">0.15</td><td rowspan="5">0.15</td><td rowspan="5">0.6</td><td rowspan="5">0.15</td><td rowspan="5">0.15</td></tr>
<tr><td>anaerobic digestion</td></tr>
<tr><td>heat drying</td></tr>
<tr><td>incineration</td></tr>
<tr><td>combined heat and power</td></tr>
<tr><td>hydrothermal liquefaction</td><td rowspan="5">0.3</td><td rowspan="5">0.3</td><td rowspan="5">0.3</td><td rowspan="5">0.3</td><td rowspan="5">0.9</td><td rowspan="5">0.3</td><td rowspan="5">0.3</td><td rowspan="5">1.2</td><td rowspan="5">0.3</td><td rowspan="5">0.3</td></tr>
<tr><td>hydrothermal alkaline treatment</td></tr>
<tr><td>supercritical water oxidation</td></tr>
<tr><td>pyrolysis</td></tr>
<tr><td>gasification</td></tr>
<tr><td colspan="11">labor costs</td></tr>
<tr><td>[MM$·FTE^{-1}·year^{-1}]</td><td>0.22</td><td>0.10</td><td>0.086</td><td>0.086</td><td>0.067</td><td>0.057</td><td>0.057</td><td>0.068</td><td>0.043</td><td>0.057</td></tr>
</table>

Table S14. Estimated labor costs for 40 wastewater residual solids management pathways.

**code**	**full-time equivalent (FTE)**	**fixed labor cost** [MM$·year^{-1}]	**variable labor cost** [MM$·year^{-1}]
C1	0.15	0.0707	0.100
C2	0.20	0.0942	0.133
C3	0.20	0.0942	0.133
C4	0.20	0.0942	0.133
C5	0.30	0.141	0.199
C6	0.30	0.141	0.199
C7	0.40	0.188	0.266
C8	0.30	0.141	0.199
C9	0.30	0.141	0.199
C10	0.35	0.165	0.233
C11	0.45	0.212	0.299
C12	0.45	0.212	0.299
C13	0.55	0.259	0.366
C14	0.30	0.141	0.199
C15	0.30	0.141	0.199
C16	0.35	0.165	0.233
C17	0.45	0.212	0.299
C18	0.45	0.212	0.299
C19	0.55	0.259	0.366
C20	0.45	0.212	0.299
C21	0.45	0.212	0.299
C22	0.50	0.236	0.332
C23	0.60	0.283	0.399
C24	0.60	0.283	0.399
C25	0.70	0.330	0.465
T1	0.55	0.259	0.366
T2	0.55	0.259	0.366
T3	0.40	0.188	0.266
T4	0.70	0.330	0.465
T5	0.70	0.330	0.465
T6	0.70	0.330	0.465
T7	0.70	0.330	0.465
T8	0.55	0.259	0.366
T9	0.85	0.400	0.565
T10	0.85	0.400	0.565
T11	0.70	0.330	0.465
T12	0.70	0.330	0.465
T13	0.55	0.259	0.366
T14	0.85	0.400	0.565
T15	0.85	0.400	0.565

S3.5. Nth-of-a-kind assumptions

TEA was initially performed under nth-of-a-kind assumptions, which exclude additional cost associated with technological immaturity. The discounted cash flow analysis was conducted to estimate the WWRS management cost. Specifically, initial capital costs were distributed over the plant's lifetime with a discount rate to account for the time value of money. Operation and maintenance costs were determined with assumptions listed in **Table S15**.

Table S15. Assumptions for the discounted cash flow analysis.

parameters	values
internal rate of return	3%
project lifetime	30 years
income tax	30%
yearly operating hours	7,884
construction schedule	3 years (8% 1st year, 60% 2nd year, 32% 3rd year)
startup month	6
startup fixed operating cost fraction	1
startup sales fraction	0.5
startup volatile operating cost fraction	0.75
working capital over fixed capital investment (FCI)	5%
finance interest	3%
finance years	10
finance fraction	0.6
warehouse	4% of installed equipment cost
site development	9% of inside battery limits (ISBL)
additional piping	4.5% of ISBL
proratable costs	10% of total direct cost (TDC)
field expenses	10% of TDC
home office and construction	20% of TDC
project Contingency	10% of TDC
other indirect costs	10% of TDC
labor burden	90%
property insurance and taxes	0.7% of FCI
maintenance	3% of total indirect cost
steam power depreciation	MACRS20

S3.6. First-of-a-kind assumptions

To estimate first-of-a-kind costs for thermochemical technologies, TEA results under nth-of-a-kind assumptions were adjusted using the RAND equations (**Eqs. S4 and S5**)[95]. The parameter values of the RAND equations for each thermochemical technologies are summarized in **Table S16**.

$$CGF = 1.12 - 0.00297 \times PNW - 0.0213 \times IMP - 0.0114 \times CPX + 0.00111 \times ICL - C \times PDN \quad \textbf{Eq. S4}$$

CGF: cost growth factor, the total capital investment ratio between pioneer plants and nth plants
PNW: percentage of capital cost of commercially undemonstrated equipment

IMP: impurity buildup and corrosion issues, ranging from 0 to 5
CPX: number of continuously linked process steps, ranging from 1 to 11
ICL: percentage of pre-startup personnel, inventory, and land purchase costs
C: 0.0401 for commercial processes; 0.0636 for research- and development-intensive processes
PDN: level of site-specific information and engineering in the estimate, ranging from 2 to 8

$$PPF = 85.8 - 9.69 \times NSS + 0.33 \times BLQ - 4.12 \times WAS - 17.9 \times SLS \qquad \textbf{Eq. S5}$$

PPF: plant performance factor, actual average production in months 7 to 12 after start-up as a percentage of plant design capacity production
NSS: number of processes incorporating commercially unproven technologies
BLQ: percentage of heat and mass balance equations based on actual data from prior plants
WAS: difficulties with waste handling encountered during development, ranging from 0 to 5
SLS: 0 for no solids handling, 1 for solids handling

Table S16. RAND equation variable values for thermochemical technologies. Abbreviations: hydrothermal liquefaction (HTL), hydrothermal alkaline treatment (HALT), catalytic hydrothermal gasification (CHG), supercritical water oxidation (SCWO). The explanation on RAND equation variables can be found in **Section S3.6**.

variable	HTL[96]	HALT[a]	CHG[a]	SCWO[a]	pyrolysis[97]	gasification[66]
			cost growth			
PNW	41	55	50	50	19	19
IMP	4	5	4	5	4	4
CPX	6	7	6	6	6	6
ICL	33	33	33	33	33	33
C	0.06361	0.06361	0.06361	0.06361	0.04011	0.04011
PDN	5	6	5	5	3	3
			plant performance			
NSS	3	5	4	4	2	2
BLQ	0	0	0	0	0	0
WAS	2	2	2	2	2	2
SLS	1	1	0	1	1	1

[a] Values in the column were estimated.

S4. Life cycle assessment (LCA)

The environmental impacts of WWRS management system were assessed based on material and utility requirements, product outputs, and direct emissions during the operation stage. LCA was conducted with a focus on the carbon intensity (CI; a.k.a. global warming potential, GWP) to characterize the system-level GHG emissions. The CI data are provided in **Table S17**.

Table S17. Life cycle assessment inventory data. A data source of 'Ecoinvent' indicates the data was obtained from Ecoinvent v3.11[70] with 'IPCC 2021, climate change: total (excl. biogenic CO_2), global warming potential (GWP_{100})' as the life cycle impact assessment methodology. Abbreviations: carbon intensity (CI), catalytic hydrothermal gasification (CHG), Biosolids Emissions Assessment Model version 2024 (BEAM*2024), Intergovernmental Panel on Climate Change (IPCC).

**item**	**unit**	**source**	**CI** [kg CO_2e]
market for polyacrylamide	kg	Ecoinvent[70]	proprietary
market for quicklime, milled, loose	kg	Ecoinvent[70]	proprietary
market for sawdust, wet, measured as dry mass	kg	Ecoinvent[70]	proprietary
market for diesel	kg	Ecoinvent[70]	proprietary
market for petroleum	kg	Ecoinvent[70]	proprietary
biosolids - carbon sequestration	kg	BEAM*2024[52,53]	0.4475
hydrochar - carbon sequestration	kg	BEAM*2024[52,53]	0.745
biochar - carbon sequestration	kg	BioFlux[98]	2
market for coal tar	kg	Ecoinvent[70]	proprietary
CHG catalyst (based on Snowden-Swan *et al.*, 2016[99])	kg	Ecoinvent[70]	proprietary
market for sodium hydroxide, without water, in 50% solution state	kg	Ecoinvent[70]	proprietary
market for hydrochloric acid, without water, in 30% solution state	kg	Ecoinvent[70]	proprietary
CH_4	kg	IPCC[100]	27
N_2O	kg	IPCC[100]	273
nitrogen (as a nutrient)	kg	BEAM*2024[52,53]	3
phosphorus (as a nutrient)	kg	BEAM*2024[52,53]	2
market for inert waste, for final disposal	kg	Ecoinvent[70]	proprietary
market for water, deionised (deionized)	kg	Ecoinvent[70]	proprietary
cooling tower chemical (scaled by its relative price to water)	kg	Ecoinvent[70]	proprietary
market for electricity, low voltage	kWh	Ecoinvent[70]	proprietary
market for cooling energy	MJ	Ecoinvent[70]	proprietary
market for heat, from steam, in chemical industry	MJ	Ecoinvent[70]	proprietary
market for heat, district or industrial, natural gas	MJ	Ecoinvent[70]	proprietary
market for natural gas, low pressure	m^3	Ecoinvent[70]	proprietary
market for transport, freight, lorry, unspecified	tonne·km	Ecoinvent[70]	proprietary

S5. Supplementary results

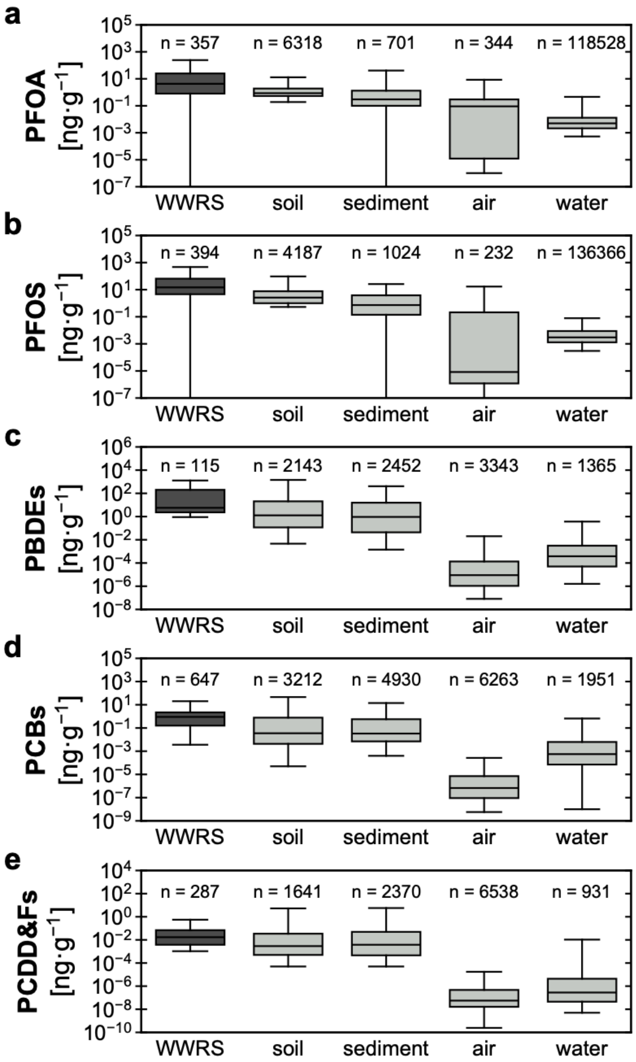

Fig. S1. Global concentrations of (a) perfluorooctanoic acid (PFOA), (b) perfluorooctane sulfonate (PFOS), (c) polybrominated diphenyl ethers (PBDEs), (d) polychlorinated biphenyls (PCBs), and (e) polychlorinated dibenzo-*p*-dioxins and polychlorinated dibenzofurans (PCDD&Fs). Concentrations are shown in darker gray for wastewater residual solids (WWRS) and in lighter gray for other environmental compartments. Concentrations of contaminants in WWRS, soil, and sediment are on a dry-weight basis. Whiskers, boxes, and midlines of box plots represent 5th and 95th percentiles, 25th and 75th percentiles, and 50th percentile, respectively. Source data are provided as a Source Data file.

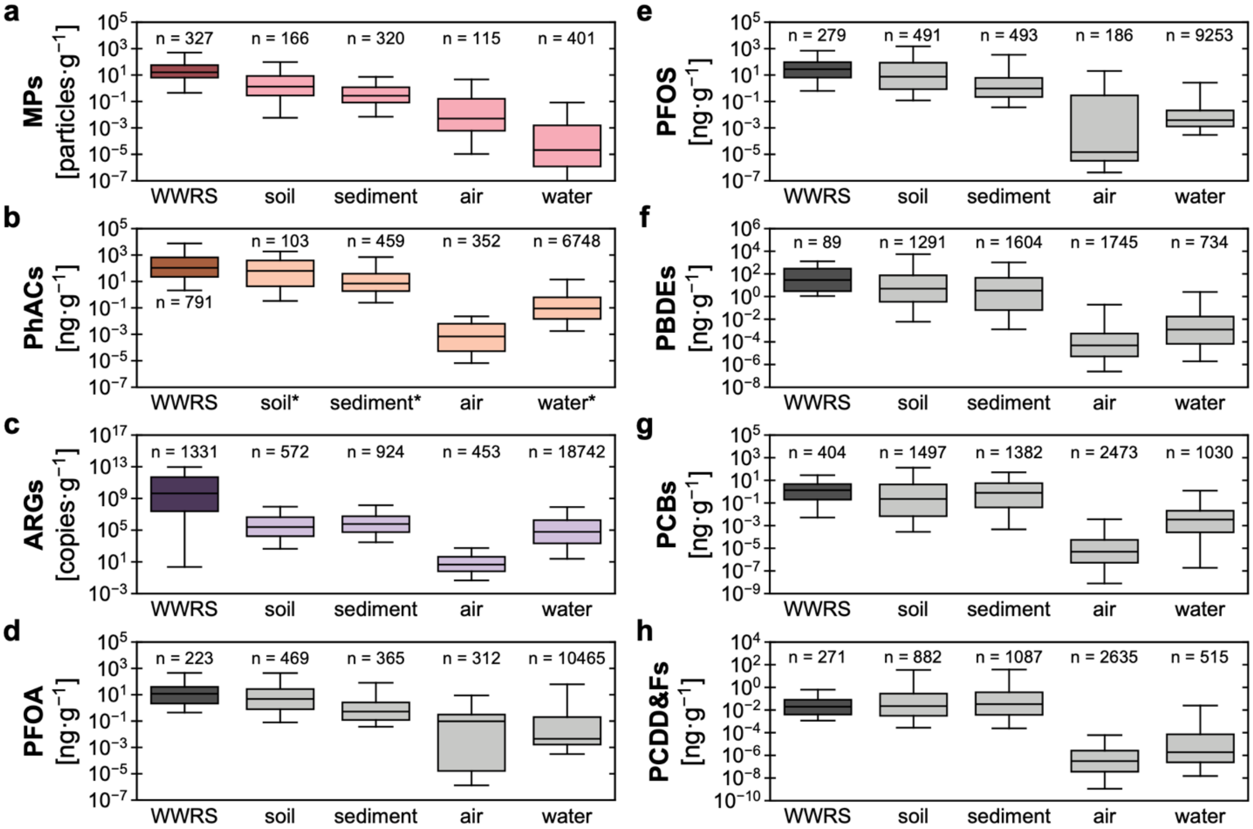

Fig. S2. Global concentrations of (a) microplastics (MPs, red), (b) pharmaceuticals (PhACs, orange), (c) antibiotic resistance genes (ARGs, purple), (d) perfluorooctanoic acid (PFOA, gray), (e) perfluorooctane sulfonate (PFOS, gray), (f) polybrominated diphenyl ethers (PBDEs, gray), (g) polychlorinated biphenyls (PCBs, gray), and (h) polychlorinated dibenzo-*p*-dioxins and polychlorinated dibenzofurans (PCDD&Fs, gray) after removing identical values within each contaminant–compartment pair. Concentrations are shown in darker color schemes for wastewater residual solids (WWRS) and in lighter color schemes for other environmental compartments. Concentrations of contaminants in WWRS, soil, and sediment are on a dry-weight basis. Whiskers, boxes, and midlines of box plots represent 5th and 95th percentiles, 25th and 75th percentiles, and 50th percentile, respectively. Source data are provided as a Source Data file.

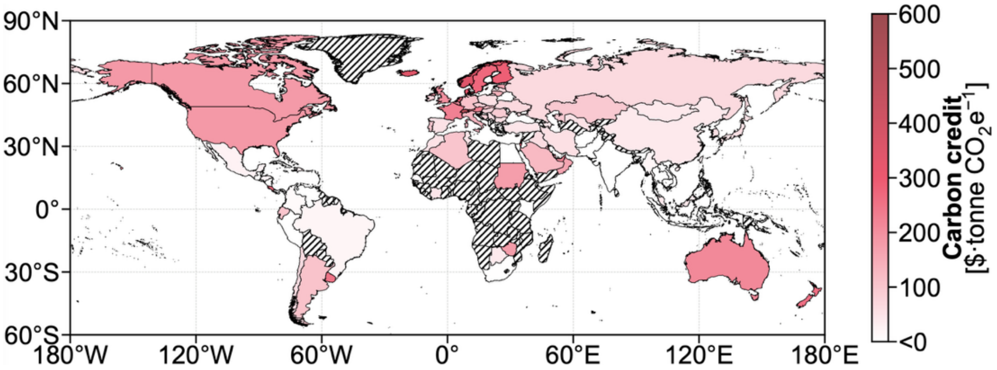

Fig. S3. Country-level average carbon credit needed for conventional and thermochemical wastewater residual solids management to achieve cost parity. Darker red indicates higher required credits. Source data are provided as a Source Data file.

References

(1) Li, Y.; Bhagwat, S. S.; Cortés-Peña, Y. R.; Ki, D.; Rao, C. V.; Jin, Y.-S.; Guest, J. S. Sustainable Lactic Acid Production from Lignocellulosic Biomass. *ACS Sustain. Chem. Eng.* **2021**, *9* (3), 1341–1351. https://doi.org/10.1021/acssuschemeng.0c08055.

(2) Feng, J.; Li, Y.; Strathmann, T. J.; Guest, J. S. Characterizing the Opportunity Space for Sustainable Hydrothermal Valorization of Wet Organic Wastes. *Environ. Sci. Technol.* **2024**, *58* (5), 2528–2541. https://doi.org/10.1021/acs.est.3c07394.

(3) Feng, J.; Strathmann, T. J.; Guest, J. S. Hydrothermal-Based Wastewater Solids Management for Targeted Resource Recovery and Decarbonization in the Contiguous U.S. *Environ. Sci. Technol.* **2025**, *59* (38), 20389–20400. https://doi.org/10.1021/acs.est.5c07190.

(4) El Abbadi, S. H.; Feng, J.; Hodson, A. R.; Amouamouha, M.; Busse, M. M.; Polcuch, C.; Zhou, P.; Macknick, J.; Guest, J. S.; Stokes-Draut, J. R.; Dunn, J. B. Benchmarking Greenhouse Gas Emissions from US Wastewater Treatment for Targeted Reduction. *Nat. Water* **2025**, *3* (10), 1133–1143. https://doi.org/10.1038/s44221-025-00485-w.

(5) Wang, Z.; Feng, J.; Shi, B.; Mendoza, J. A.; Zhang, X.; Trousdale, N.; Cusick, R. D.; Yee, S.; Guest, J. S. The Potential of Thermomechanical and Thermochemical Processes to Enable Sustainable Household Sanitation. *Environ. Sci. Technol.* **2026**, *60* (8), 6227–6238. https://doi.org/10.1021/acs.est.5c15639.

(6) Boucher, J.; Friot, D. *Primary Microplastics in the Oceans: A Global Evaluation of Sources*; IUCN International Union for Conservation of Nature, 2017. https://doi.org/10.2305/IUCN.CH.2017.01.en.

(7) Ryberg, M. W.; Laurent, A.; Hauschild, M. *Mapping of Global Plastics Value Chain and Plastics Losses to the Environment (With a Particular Focus on Marine Environment)*; United Nations Environment Programme: Nairobi, Kenya, 2018. https://www.unep.org/resources/report/mapping-global-plastics-value-chain-and-plastics-losses-environment-particular (accessed 2025-09-22).

(8) Wang, R.; Balkanski, Y.; Boucher, O.; Bopp, L.; Chappell, A.; Ciais, P.; Hauglustaine, D.; Peñuelas, J.; Tao, S. Sources, Transport and Deposition of Iron in the Global Atmosphere. *Atmospheric Chem. Phys.* **2015**, *15* (11), 6247–6270. https://doi.org/10.5194/acp-15-6247-2015.

(9) *Fact #902: December 7, 2015 Rural versus Urban Vehicle Miles of Travel by State*. Energy.gov. https://www.energy.gov/eere/vehicles/fact-902-december-7-2015-rural-versus-urban-vehicle-miles-travel-state (accessed 2025-10-05).

(10) Husqvarna Urban Green Space Insights. *Urban Green Space Report 2024 - How Green Are Cities?*; 2024.

(11) US EPA, O. *Urbanization - Wastewater Inputs*. https://www.epa.gov/caddis/urbanization-wastewater-inputs (accessed 2025-10-05).

(12) Morita, H. *Present Status of Combined Sewer Overflow and New CSO Control Policy in JAPAN*; Ministry Of Land, Infrastructure And Transport.

(13) Park, S. Odor Characteristics and Concentration of Malodorous Chemical Compounds Emitted from a Combined Sewer System in Korea. *Atmosphere* **2020**, *11* (6), 667. https://doi.org/10.3390/atmos11060667.

(14) Harley-Nyang, D.; Memon, F. A.; Jones, N.; Galloway, T. Investigation and Analysis of Microplastics in Sewage Sludge and Biosolids: A Case Study from One Wastewater

Treatment Works in the UK. *Sci. Total Environ.* **2022**, *823*, 153735. https://doi.org/10.1016/j.scitotenv.2022.153735.
(15) Koutnik, V. S.; Alkidim, S.; Leonard, J.; DePrima, F.; Cao, S.; Hoek, E. M. V.; Mohanty, S. K. Unaccounted Microplastics in Wastewater Sludge: Where Do They Go? *ACS EST Water* **2021**, *1* (5), 1086–1097. https://doi.org/10.1021/acsestwater.0c00267.
(16) US EPA, O. *Urbanization - Wastewater Inputs*. https://www.epa.gov/caddis/urbanization-wastewater-inputs (accessed 2025-11-17).
(17) Saddiqi, M. M.; Zhao, W.; Cotterill, S.; Dereli, R. K. Smart Management of Combined Sewer Overflows: From an Ancient Technology to Artificial Intelligence. *WIREs Water* **2023**, *10* (3), e1635. https://doi.org/10.1002/wat2.1635.
(18) Park, S. Odor Characteristics and Concentration of Malodorous Chemical Compounds Emitted from a Combined Sewer System in Korea. *Atmosphere* **2020**, *11* (6), 667. https://doi.org/10.3390/atmos11060667.
(19) Cook, S. M.; VanDuinen, B. J.; Love, N. G.; Skerlos, S. J. Life Cycle Comparison of Environmental Emissions from Three Disposal Options for Unused Pharmaceuticals. *Environ. Sci. Technol.* **2012**, *46* (10), 5535–5541. https://doi.org/10.1021/es203987b.
(20) Abdallah, M.; Bethäuser, J.; Tettenborn, F.; Hein, A.; Hamann, M. Pharmaceutical Consumption in Human and Veterinary Medicine in Germany: Potential Environmental Challenges. *Front. Environ. Sci.* **2024**, *12*. https://doi.org/10.3389/fenvs.2024.1443935.
(21) *Veterinary Pharmaceuticals Market Size, Share | 2022 - 2027*. MarketsandMarkets. https://www.marketsandmarkets.com/Market-Reports/veterinary-pharmaceuticals-market-243381376.html (accessed 2025-09-29).
(22) Makki, M.; Akmal Shafie, A.; Awaisu, A.; Hussain, R.; Al Hail, M.; ElMotasim, W. M.; Mohamed Ali Taha, M. Y.; Abdoun, E.; Al-Khuzaei, N. M. J.; Salama, G.; Pallivalapila, A.; El Kassem, W.; Thomas, B. Patients' Knowledge, Attitude, and Practices toward Unused Medications in Qatar: A Cross-Sectional Survey. *Heliyon* **2024**, *10* (12), e31931. https://doi.org/10.1016/j.heliyon.2024.e31931.
(23) Watkins, S.; Barnett, J.; Standage, M.; Kasprzyk-Hordern, B.; Barden, R. Household Disposal of Pharmaceuticals: Attitudes and Risk Perception in a UK Sample. *J. Mater. Cycles Waste Manag.* **2022**, *24* (6), 2455–2469. https://doi.org/10.1007/s10163-022-01494-7.
(24) Quadra, G. R.; Silva, P. S. A.; Paranaíba, J. R.; Josué, I. I. P.; Souza, H.; Costa, R.; Fernandez, M.; Vilas-Boas, J.; Roland, F. Investigation of Medicines Consumption and Disposal in Brazil: A Study Case in a Developing Country. *Sci. Total Environ.* **2019**, *671*, 505–509. https://doi.org/10.1016/j.scitotenv.2019.03.334.
(25) Sonowal, S.; Desai, C.; Kapadia, J. D.; Desai, M. K. A Survey of Knowledge, Attitude, and Practice of Consumers at a Tertiary Care Hospital Regarding the Disposal of Unused Medicines. *J. Basic Clin. Pharm.* **2016**, *8* (1), 4–7. https://doi.org/10.4103/0976-0105.195079.
(26) OECD. *Management of Pharmaceutical Household Waste - Limiting Environmental Impacts of Unused or Expired Medicine*; 2022. https://www.oecd.org/en/publications/management-of-pharmaceutical-household-waste_3854026c-en.html (accessed 2025-09-29).
(27) Bound, J. P.; Voulvoulis, N. Household Disposal of Pharmaceuticals as a Pathway for Aquatic Contamination in the United Kingdom. *Environ. Health Perspect.* **2005**. https://doi.org/10.1289/ehp.8315.

(28) Yohannes, L.; Tegegne, A. A.; Genet, G.; Workie Limenh, L.; Mohammed Seid, A.; Alemayehu, T. T.; Ayenew, W.; Simegn, W. Disposal Practice and Determinants of Unused Medicines among the General Public in Gondar City, Northwest Ethiopia. *Front. Public Health* **2025**, *13*. https://doi.org/10.3389/fpubh.2025.1516809.
(29) Tong, A. Y. C.; Peake, B. M.; Braund, R. Disposal Practices for Unused Medications around the World. *Environ. Int.* **2011**, *37* (1), 292–298. https://doi.org/10.1016/j.envint.2010.10.002.
(30) Toe, J.; Orok, E.; Erah, P. Assessment of Knowledge and Disposal Practices of Unused and Expired Household Medicines in a Community in Liberia. *Explor. Res. Clin. Soc. Pharm.* **2023**, *12*, 100369. https://doi.org/10.1016/j.rcsop.2023.100369.
(31) Patel, M.; Kumar, R.; Kishor, K.; Mlsna, T.; Pittman, C. U. Jr.; Mohan, D. Pharmaceuticals of Emerging Concern in Aquatic Systems: Chemistry, Occurrence, Effects, and Removal Methods. *Chem. Rev.* **2019**, *119* (6), 3510–3673. https://doi.org/10.1021/acs.chemrev.8b00299.
(32) Watkins, S.; Barnett, J.; Standage, M.; Kasprzyk-Hordern, B.; Barden, R. Household Disposal of Pharmaceuticals: Attitudes and Risk Perception in a UK Sample. *J. Mater. Cycles Waste Manag.* **2022**, *24* (6), 2455–2469. https://doi.org/10.1007/s10163-022-01494-7.
(33) Organisation for Economic Co-operation and Development. *Management of Pharmaceutical Household Waste - Limiting Environmental Impacts of Unused or Expired Medicine*; 2022.
(34) Bound, J. P.; Voulvoulis, N. Household Disposal of Pharmaceuticals as a Pathway for Aquatic Contamination in the United Kingdom. *Environ. Health Perspect.* **2005**, *113* (12), 1705–1711. https://doi.org/10.1289/ehp.8315.
(35) Tong, A. Y. C.; Peake, B. M.; Braund, R. Disposal Practices for Unused Medications around the World. *Environ. Int.* **2011**, *37* (1), 292–298. https://doi.org/10.1016/j.envint.2010.10.002.
(36) Quadra, G. R.; Silva, P. S. A.; Paranaíba, J. R.; Josué, I. I. P.; Souza, H.; Costa, R.; Fernandez, M.; Vilas-Boas, J.; Roland, F. Investigation of Medicines Consumption and Disposal in Brazil: A Study Case in a Developing Country. *Sci. Total Environ.* **2019**, *671*, 505–509. https://doi.org/10.1016/j.scitotenv.2019.03.334.
(37) Sonowal, S.; Desai, C.; Kapadia, J. D.; Desai, M. K. A Survey of Knowledge, Attitude, and Practice of Consumers at a Tertiary Care Hospital Regarding the Disposal of Unused Medicines. *J. Basic Clin. Pharm.* **2016**, *8* (1), 4–7. https://doi.org/10.4103/0976-0105.195079.
(38) Yohannes, L.; Tegegne, A. A.; Genet, G.; Workie Limenh, L.; Mohammed Seid, A.; Alemayehu, T. T.; Ayenew, W.; Simegn, W. Disposal Practice and Determinants of Unused Medicines among the General Public in Gondar City, Northwest Ethiopia. *Front. Public Health* **2025**, *13*. https://doi.org/10.3389/fpubh.2025.1516809.
(39) Toe, J.; Orok, E.; Erah, P. Assessment of Knowledge and Disposal Practices of Unused and Expired Household Medicines in a Community in Liberia. *Explor. Res. Clin. Soc. Pharm.* **2023**, *12*, 100369. https://doi.org/10.1016/j.rcsop.2023.100369.
(40) Pruden, A.; Larsson, D. G. J.; Amézquita, A.; Collignon, P.; Brandt, K. K.; Graham, D. W.; Lazorchak, J. M.; Suzuki, S.; Silley, P.; Snape, J. R.; Topp, E.; Zhang, T.; Zhu, Y.-G. Management Options for Reducing the Release of Antibiotics and Antibiotic Resistance

Genes to the Environment. *Environ. Health Perspect.* **2013**, *121* (8), 878–885. https://doi.org/10.1289/ehp.1206446.
(41) Wu, D.; Van Goethem, M. W.; Graham, D. W.; Zhang, X.; Li, Z.; Shi, G. Antarctic Environmental Resistomes Closely Associated with Human and Animal Waste Releases. *Environ. Sci. Technol.* **2025**. https://doi.org/10.1021/acs.est.5c06023.
(42) Dattani, S.; Spooner, F.; Ritchie, H.; Roser, M. Antibiotics and Antibiotic Resistance. *Our World Data* **2024**.
(43) The Wellcome Trust. *Initiatives for Addressing Antimicrobial Resistance in the Environment - Current Situation and Challenges*; The Wellcome Trust, 2018. https://cms.wellcome.org/sites/default/files/antimicrobial-resistance-environment-report.pdf (accessed 2025-10-04).
(44) Liu, Z.; Lu, Y.; Wang, P.; Wang, T.; Liu, S.; Johnson, A. C.; Sweetman, A. J.; Baninla, Y. Pollution Pathways and Release Estimation of Perfluorooctane Sulfonate (PFOS) and Perfluorooctanoic Acid (PFOA) in Central and Eastern China. *Sci. Total Environ.* **2017**, *580*, 1247–1256. https://doi.org/10.1016/j.scitotenv.2016.12.085.
(45) *PFAS Regulation around the world.* Antea Group. https://int.anteagroup.com/news-and-media/blog/pfas-regulation-around-the-world (accessed 2025-11-17).
(46) *Quantitative Sustainable Design (QSD) Group*. GitHub. https://github.com/QSD-Group (accessed 2025-12-04).
(47) Li, Y.; Zhang, X.; Morgan, V. L.; Lohman, H. A. C.; Rowles, L. S.; Mittal, S.; Kogler, A.; Cusick, R. D.; Tarpeh, W. A.; Guest, J. S. QSDsan: An Integrated Platform for Quantitative Sustainable Design of Sanitation and Resource Recovery Systems. *Environ. Sci. Water Res. Technol.* **2022**, 10.1039.D2EW00455K. https://doi.org/10.1039/D2EW00455K.
(48) *BioSTEAM Development Group*. GitHub. https://github.com/BioSTEAMDevelopmentGroup (accessed 2025-12-04).
(49) Cortes-Peña, Y.; Kumar, D.; Singh, V.; Guest, J. S. BioSTEAM: A Fast and Flexible Platform for the Design, Simulation, and Techno-Economic Analysis of Biorefineries under Uncertainty. *ACS Sustain. Chem. Eng.* **2020**, *8* (8), 3302–3310. https://doi.org/10.1021/acssuschemeng.9b07040.
(50) ASMARKETING. *Gravity vs. Mechanical Sludge Thickening: Which One Wins?*. Sludge Dryer. https://sludgedryer.in/gravity-vs-mechanical-sludge-thickener-comparison/ (accessed 2025-07-06).
(51) Seider, W. D.; Lewin, D. R.; Seader, J. D.; Widagdo, S.; Gani, R.; Ng, K. M. Design Optimization. In *Product and Process Design Principles*; Wiley, 2017.
(52) Brown, S.; Beecher, N.; Carpenter, A. Calculator Tool for Determining Greenhouse Gas Emissions for Biosolids Processing and End Use. *Environ. Sci. Technol.* **2010**, *44* (24), 9509–9515. https://doi.org/10.1021/es101210k.
(53) North East Biosolids and Residuals Association (NEBRA), Northern Tilth LLC, and Northwest Biosolids. Estimating Greenhouse Gas Emissions from Biosolids Management. BEAM*2024 Spreadsheet Model and Supporting Information, 2024. https://www.BiosolidsGHGs.org (accessed 2025-06-16).
(54) Shoener, B. D.; Zhong, C.; Greiner, A. D.; Khunjar, W. O.; Hong, P.-Y.; Guest, J. S. Design of Anaerobic Membrane Bioreactors for the Valorization of Dilute Organic Carbon Waste Streams. *Energy Environ. Sci.* **2016**, *9* (3), 1102–1112. https://doi.org/10.1039/C5EE03715H.

(55) *Bio-Sludge Anaerobic Digester Tank for Industrial Wastewater Treatment Plant: 30 Years of Reliable Service*. Shijiazhuang Zhengzhong Technology Co., Ltd. https://www.cectanks.com/quality-8877275-bio-sludge-anaerobic-digester-tank-for-industrial-wastewater-treatment-plant-30-years-of-reliable-se (accessed 2025-07-06).
(56) *Regular Maintenance of Sludge Dewatering Machines: A Key to Prolonging Machine Life*. Sludge treatment | AMCON INC. https://www.amcon-jp.com/useful/use-sludge-treatment/8284/ (accessed 2025-07-06).
(57) Williford, C.; Chen, W.-Y.; Shamas, N. K.; Wang, L. K. Lime Stabilization. In *Biosolids Treatment Processes*; Wang, L. K., Shammas, N. K., Hung, Y.-T., Eds.; Humana Press: Totowa, NJ, 2007; pp 207–241. https://doi.org/10.1007/978-1-59259-996-7_7.
(58) Stramer, Y.; Brenner, A.; Cohen, S. B.; Oron, G. Selection of a Multi-Stage System for Biosolids Management Applying Genetic Algorithm. *Environ. Sci. Technol.* **2010**, *44* (14), 5503–5508. https://doi.org/10.1021/es902981t.
(59) Hao, X.; Chen, Q.; van Loosdrecht, M. C. M.; Li, J.; Jiang, H. Sustainable Disposal of Excess Sludge: Incineration without Anaerobic Digestion. *Water Res.* **2020**, *170*, 115298. https://doi.org/10.1016/j.watres.2019.115298.
(60) *Sludge Incineration Kiln*. CEMENTL. https://www.cementl.com/product/sludge-incineration-kiln/ (accessed 2025-07-06).
(61) Gergel, I. *Cost of incineration plant*. Waste To Energy International. https://wteinternational.com/news/cost-of-incineration-plant/ (accessed 2025-08-06).
(62) Jones, S. B.; Zhu, Y.; Anderson, D. B.; Hallen, R. T.; Elliott, D. C.; Schmidt, A. J.; Albrecht, K. O.; Hart, T. R.; Butcher, M. G.; Drennan, C.; Snowden-Swan, L. J.; Davis, R.; Kinchin, C. *Process Design and Economics for the Conversion of Algal Biomass to Hydrocarbons: Whole Algae Hydrothermal Liquefaction and Upgrading*; PNNL--23227, 1126336; 2014; p PNNL--23227, 1126336. https://doi.org/10.2172/1126336.
(63) Qiu, Y.; Zhang, F.; Yuan, Y.; Zhao, Y.; Liu, Y.; Rong, W. Thermodynamic and Economic Comparisons of Supercritical Water Oxidation and Gasification of Oily Sludge under Hydrothermal Flames. *Int. J. Hydrog. Energy* **2024**, *85*, 571–585. https://doi.org/10.1016/j.ijhydene.2024.08.362.
(64) Shoaib Ahmed Khan, M.; Grioui, N.; Halouani, K.; Benelmir, R. Techno-Economic Analysis of Production of Bio-Oil from Catalytic Pyrolysis of Olive Mill Wastewater Sludge with Two Different Cooling Mechanisms. *Energy Convers. Manag. X* **2022**, *13*, 100170. https://doi.org/10.1016/j.ecmx.2021.100170.
(65) Clack, K.; Rajagopal, D.; Hoek, E. M. V. Life Cycle and Techno-Economic Assessment of Bioresource Production from Wastewater. *Npj Clean Water* **2024**, *7* (1), 1–17. https://doi.org/10.1038/s41545-024-00314-9.
(66) Swanson, R. M.; Platon, A.; Satrio, J. A.; Brown, R. C.; Hsu, D. D. *Techno-Economic Analysis of Biofuels Production Based on Gasification*; 2010.
(67) Maryland Energy Administration. Combined Heat and Power (CHP) Resource Guide, 2023. https://energy.maryland.gov/business/Documents/MEA%20CHP%20Resource%20Guide%202023.pdf (accessed 2025-07-06).

(68) *Cooling Tower Lifespan: Factors, Failures & Fixes - New Cooling Tower Construction, Parts, Maintenance, Upgrades*. https://h2ocooling.com/blog/cooling-tower-lifespan-factors-failures-fixes/ (accessed 2025-08-11).
(69) Humbird, D.; Davis, R.; Tao, L.; Kinchin, C.; Hsu, D.; Aden, A.; Schoen, P.; Lukas, J.; Olthof, B.; Worley, M.; Sexton, D.; Dudgeon, D. *Process Design and Economics for Biochemical Conversion of Lignocellulosic Biomass to Ethanol: Dilute-Acid Pretreatment and Enzymatic Hydrolysis of Corn Stover*; NREL/TP-5100-47764, 1013269; 2011; p NREL/TP-5100-47764, 1013269. https://doi.org/10.2172/1013269.
(70) Ecoinvent v3.11 Database. Swiss Centre for Life Cycle Inventories (accessed 2025-11-06).
(71) Wang, Q.; Yuan, Z. Enhancing Aerobic Digestion of Full-Scale Waste Activated Sludge Using Free Nitrous Acid Pre-Treatment. *RSC Adv.* **2015**, *5* (25), 19128–19134. https://doi.org/10.1039/C4RA17215A.
(72) US EPA, O. *Waste Reduction Model*. https://www.epa.gov/waste-reduction-model (accessed 2025-11-17).
(73) Leow, S.; Witter, J. R.; Vardon, D. R.; Sharma, B. K.; Guest, J. S.; Strathmann, T. J. Prediction of Microalgae Hydrothermal Liquefaction Products from Feedstock Biochemical Composition. *Green Chem.* **2015**, *17* (6), 3584–3599. https://doi.org/10.1039/C5GC00574D.
(74) Li, Y.; Leow, S.; Fedders, A. C.; Sharma, B. K.; Guest, J. S.; Strathmann, T. J. Quantitative Multiphase Model for Hydrothermal Liquefaction of Algal Biomass. *Green Chem.* **2017**, *19* (4), 1163–1174. https://doi.org/10.1039/C6GC03294J.
(75) Leow, S.; Shoener, B. D.; Li, Y.; DeBellis, J. L.; Markham, J.; Davis, R.; Laurens, L. M. L.; Pienkos, P. T.; Cook, S. M.; Strathmann, T. J.; Guest, J. S. A Unified Modeling Framework to Advance Biofuel Production from Microalgae. *Environ. Sci. Technol.* **2018**, *52* (22), 13591–13599. https://doi.org/10.1021/acs.est.8b03663.
(76) Muangrat, R.; Onwudili, J. A.; Williams, P. T. Reaction Products from the Subcritical Water Gasification of Food Wastes and Glucose with NaOH and H2O2. *Bioresour. Technol.* **2010**, *101* (17), 6812–6821. https://doi.org/10.1016/j.biortech.2010.03.114.
(77) Pelagalli, V.; Langone, M.; Matassa, S.; Race, M.; Tuffi, R.; Papirio, S.; Lens, P. N. L.; Lazzazzara, M.; Frugis, A.; Petta, L.; Esposito, G. Pyrolysis of Municipal Sewage Sludge: Challenges, Opportunities and New Valorization Routes for Biochar, Bio-Oil, and Pyrolysis Gas. *Environ. Sci. Water Res. Technol.* **2024**, *10* (10), 2282–2312. https://doi.org/10.1039/D4EW00278D.
(78) Lumley, N. P. G.; Ramey, D. F.; Prieto, A. L.; Braun, R. J.; Cath, T. Y.; Porter, J. M. Techno-Economic Analysis of Wastewater Sludge Gasification: A Decentralized Urban Perspective. *Bioresour. Technol.* **2014**, *161*, 385–394. https://doi.org/10.1016/j.biortech.2014.03.040.
(79) Hao, S.; Choi, Y. J.; Deeb, R. A.; Strathmann, T. J.; Higgins, C. P. Application of Hydrothermal Alkaline Treatment for Destruction of Per- and Polyfluoroalkyl Substances in Contaminated Groundwater and Soil. *Environ. Sci. Technol.* **2022**, *56* (10), 6647–6657. https://doi.org/10.1021/acs.est.2c00654.
(80) Adegoroye, A.; Paterson, N.; Li, X.; Morgan, T.; Herod, A. A.; Dugwell, D. R.; Kandiyoti, R. The Characterisation of Tars Produced during the Gasification of Sewage Sludge in a

Spouted Bed Reactor. *Fuel* **2004**, *83* (14), 1949–1960. https://doi.org/10.1016/j.fuel.2004.04.006.
(81) Davis, R. E.; Grundl, N. J.; Tao, L.; Biddy, M. J.; Tan, E. C.; Beckham, G. T.; Humbird, D.; Thompson, D. N.; Roni, M. S. *Process Design and Economics for the Conversion of Lignocellulosic Biomass to Hydrocarbon Fuels and Coproducts: 2018 Biochemical Design Case Update; Biochemical Deconstruction and Conversion of Biomass to Fuels and Products via Integrated Biorefinery Pathways*; NREL/TP--5100-71949, 1483234; 2018; p NREL/TP--5100-71949, 1483234. https://doi.org/10.2172/1483234.
(82) Kline, K. L.; Oladosu, G. A.; Wolfe, A. K.; Perlack, R. D.; Dale, V. H.; McMahon, M. *Biofuel Feedstock Assessment for Selected Countries*; Oak Ridge National Laboratory, 2008.
(83) Davis, R.; Hawkins, T.; Coleman, A.; Gao, S.; Klein, B.; Wiatrowski, M.; Zhu, Y.; Xu, Y.; Snowden-Swan, L.; Valdez, P.; Zhang, J.; Singh, U.; Ou, L. Economic, Greenhouse Gas, and Resource Assessment for Fuel and Protein Production from Microalgae: 2022 Algae Harmonization Update. **2024**. https://doi.org/10.2172/2318964.
(84) U.S. Energy Information Administration (EIA). *U.S. Crude Oil First Purchase Price (Dollars per Barrel)*. https://www.eia.gov/dnav/pet/hist/LeafHandler.ashx?n=pet&s=f000000__3&f=m (accessed 2025-08-21).
(85) Ajaero, T. M. *How Much Does It Cost to Dispose Hazardous Waste Per Ton?*. ProfitableVenture. https://www.profitableventure.com/cost-dispose-hazardous-waste-per-ton/ (accessed 2025-08-27).
(86) Gu, X.; Yu, L.; Pang, N.; Martinez-Fernandez, J. S.; Fu, X.; Chen, S. Comparative Techno-Economic Analysis of Algal Biofuel Production via Hydrothermal Liquefaction: One Stage versus Two Stages. *Appl. Energy* **2020**, *259*, 114115. https://doi.org/10.1016/j.apenergy.2019.114115.
(87) *The Ultimate Business Guide to Biochar: Everything You Need to Know.* https://cloverly.com/blog/the-ultimate-business-guide-to-biochar-everything-you-need-to-know (accessed 2025-08-20).
(88) U.S. Energy Information Administration (EIA). *Weekly U.S. No 2 Diesel Retail Prices (Dollars per Gallon)*. https://www.eia.gov/dnav/pet/hist/LeafHandler.ashx?n=pet&s=emd_epd2d_pte_nus_dpg&f=w (accessed 2025-08-18).
(89) *Analyzing Municipal Solid Waste Landfill Tipping Fees — 2023*; The Environmental Research & Education Foundation, 2024.
(90) *BioSTEAM Development Group*. https://github.com/BioSTEAMDevelopmentGroup (accessed 2023-04-08).
(91) Marufuzzaman, M.; Ekşioğlu, S. D.; Hernandez, R. Truck versus Pipeline Transportation Cost Analysis of Wastewater Sludge. *Transp. Res. Part Policy Pract.* **2015**, *74*, 14–30. https://doi.org/10.1016/j.tra.2015.02.001.
(92) Pootakham, T.; Kumar, A. Bio-Oil Transport by Pipeline: A Techno-Economic Assessment. *Bioresour. Technol.* **2010**, *101* (18), 7137–7143. https://doi.org/10.1016/j.biortech.2010.03.136.
(93) Snowden-Swan, L. J.; Li, S.; Thorson, M. R.; Schmidt, A. J.; Cronin, D. J.; Zhu, Y.; Hart, T. R.; Santosa, D. M.; Fox, S. P.; Lemmon, T. L.; Swita, M. S. *Wet Waste Hydrothermal Liquefaction and Biocrude Upgrading to Hydrocarbon Fuels: 2022 State of Technology*;

PNNL-33622; Pacific Northwest National Lab. (PNNL), Richland, WA (United States), 2022. https://doi.org/10.2172/1897670.
(94) *Bureau of Labor Statistics Data*. Bureau of Labor Statistics. https://data.bls.gov/pdq/SurveyOutputServlet (accessed 2025-07-08).
(95) Merrow, E. W.; Phillips, K.; Myers, C. W. *Understanding Cost Growth and Performance Shortfalls in Pioneer Process Plants*; 1981. https://www.rand.org/pubs/reports/R2569.html (accessed 2025-09-18).
(96) Snowden-Swan, L.; Schmidt, A.; Fuller, C.; Anderson, D. *Hydrothermal Processing of Wastewater Solids (HYPOWERs) Project Preliminary Techno-Economic Analysis*; PNNL-36528; 2024.
(97) Wright, M. M.; Satrio, J. A.; Brown, R. C.; University, I. S. *Techno-Economic Analysis of Biomass Fast Pyrolysis to Transportation Fuels*; 2010.
(98) *What Is Biochar Carbon Removal?* https://www.bioflux.earth/blog/what-is-biochar-carbon-removal (accessed 2025-08-20).
(99) Snowden-Swan, L. J.; Spies, K. A.; Lee, G. J.; Zhu, Y. Life Cycle Greenhouse Gas Emissions Analysis of Catalysts for Hydrotreating of Fast Pyrolysis Bio-Oil. *Biomass Bioenergy* **2016**, *86*, 136–145. https://doi.org/10.1016/j.biombioe.2016.01.019.
(100) *Climate Change 2022: Impacts, Adaptation and Vulnerability. Contribution of Working Group II to the Sixth Assessment Report of the Intergovernmental Panel on Climate Change.*; Pörtner, H.-O., Roberts, D. C., Tignor, M. M. B., Poloczanska, E. S., Mintenbeck, K., Alegría, A., Craig, M., Langsdorf, S., Löschke, S., Möller, V., Okem, A., Rama, B., Eds.; 2022.
