# Supplementary material for: Thermochemical Treatment of Wastewater Residual Solids for Global Mitigation of Emerging Contaminants
Source: Nat Commun. 2026 Jun 12;17:7462. doi: 10.1038/s41467-026-74242-2 (PMC13408663; doi:10.1038/s41467-026-74242-2)
Supplement: Supplementary file 2 — Description of Additional Supplementary File [file 41467_2026_74242_MOESM2_ESM.pdf]

Description of Additional Supplementary Files

Supplementary Data 1: Raw data on emerging contaminant concentrations, emerging contaminant destruction efficiencies, country-level contextual parameters, and country -level results.
