# Supplementary material for: Thermochemical Treatment of Wastewater Residual Solids for Global Mitigation of Emerging Contaminants
Source: Nat Commun. 2026 Jun 12;17:7462. doi: 10.1038/s41467-026-74242-2 (PMC13408663; doi:10.1038/s41467-026-74242-2)
Supplement: Supplementary file 4 — Transparent Peer Review file [file 41467_2026_74242_MOESM4_ESM.pdf]

nature portfolio

Peer Review File

Thermochemical Treatment of Wastewater Residual Solids for Global Mitigation of Emerging Contaminants

Corresponding Author: Professor Jeremy Guest

This file contains all reviewer reports in order by version, followed by all author rebuttals in order by version.

Version 0:

Reviewer comments:

Reviewer #1

(Remarks to the Author)
The authors present an exciting approach to using wastewater as a centralized process to remove emerging contaminant from wastewater residual solids (WWRS).

Main comment:
I recommend the authors improve the explanation of why they did not include PFAS in the analysis along with microplastics, PhACs, and antibiotic resistant genes. On page 7, lines 141–142 the authors claim that 90% of PFAS flux into the environment is from industry and yet, we continue to hear about PFAS concentration in WRS and environmental/human exposure to PFAS from soil that was burdened and poisoned with WWRS application, and even airborne exposure from WWRS in landfills (although this could very well be from industrial waste). In addition, on p. 6 lines 102-103 the authors kind of contradict themselves regarding the importance of PFAS in WWRS, when they state that PFOA and PFOS are also concentrated in WWRS compared to the other environmental compartments analyzed in this study. I just think the non - inclusion of PFAS requires more explanation. For example, is it because PFAS requires more extreme conditions for destruction compared to other ECs? Or something else. I'm not saying the authors must include PFAS in the analysis presented in the main manuscript—just please explain better why it's not included.

Minor comments:

p. 15, line 297: what is meant by the word indices?

p. 17 lines 330–331: Please clarify the statement that "Global equity will determine the planetary significance…" It sounds weird. Are you saying that global equity is required. Or that the global north must invest in the global south? Or maybe just what's said in the last sentence of the paragraph that "thermochemical processing must be implemented equitably to restore the chemical balance of the biosphere. " So you could move the last sentence to the start of the paragraph I guess.

(Remarks on code availability)

Reviewer #2

(Remarks to the Author)
The manuscript is a generally well-written overview of emerging contaminants (microplastics, pharmaceuticals, and antibiotic resistant genes) and the use of wastewater residual solids as a removal mechanism. The authors carefully present the ecological and human health concerns surrounding the relatively underregulated release of these classes of emerging contaminants.

The manuscript will be significantly improved with some minor editing and specification of data sources and reference to supplementary data. It was not clear to this reviewer what is the source of data used in creating figures in the main manuscript. It was also difficult to differentiate between figures and tables referenced in the main manuscript and those in the Supplementary Information. Some clarification is needed.

(Remarks on code availability)

Reviewer #3

(Remarks to the Author)
The MS is very interesting, however, I was taken aback by its unconventional organization. Most MSs in scientific lietarure are arranged accorfing to a well-defined layout, helping readers to orientate themselves though the text. In particular, Materials and Methods should come after the introduction, and the section on "Thermochemical technologies for efficient EC destruction" follwoed by results and discussion.
Some comments:
L. 28 "...ECs—including microplastics (MPs)..." while MPs are certianly relevant as far as detection in many media, a more serious issue is perhaps die to nanoplastics, less detectable and possibly more pernicious (see, e.g. "Micro- and Nano-Plastics in Drinking Water: Threat or Hype? Critical State-of-the-Art Analysis of Risks and Approaches" J Xenob, 2025)
L. 35 "Source-control policies have 35 successfully reduced emissions of well-characterized industrial compounds such as per- and polyfluoroalkyl substances (PFAS)" an interesting discussion was recently published concerning PFAS emissions from WWTPs and the impact of recent regulations ("Prospects of Novel Technologies for PFAS Destruction in Water and Wastewater" Appl Sci, 2025). It appears that some of the observed reductions observed are... just observed due to regulatory loopholes.
L. 84 "... human-engineered... " pleonastic. Engineered suffices
L. 129 "...substantial share of total emissions... " I suggest "... substantial share of total waterborne emissions..." (see also the following statement "...20% ... of total global MP emissions migrate through WWRS..."
In addition, I recommend a reision to a more conventional layout before MS can be accepted-

(Remarks on code availability)
I looke dat the code webpage. I was not able to run it, but it seems compliant with general reproducibility requirements.

Version 1:

Reviewer comments:

Reviewer #1

(Remarks to the Author)
The author addressed the reviewers's comments well. I am in support of the manuscript being accepted.

(Remarks on code availability)

Reviewer #2

(Remarks to the Author)
My original comments have been addressed.

(Remarks on code availability)
I have no additonal comments.

Reviewer #3

(Remarks to the Author)
Authors have addressed satisfactorily my comments. MS can be accepted

(Remarks on code availability)

Open Access This Peer Review File is licensed under a Creative Commons Attribution 4.0 International License, which permits use, sharing, adaptation, distribution and reproduction in any medium or format, as long as you give appropriate credit to the original author(s) and the source, provide a link to the Creative Commons license, and indicate if changes were made.
In cases where reviewers are anonymous, credit should be given to 'Anonymous Referee' and the source.
The images or other third party material in this Peer Review File are included in the article's Creative Commons license, unless indicated otherwise in a credit line to the material. If material is not included in the article's Creative Commons license and your intended use is not permitted by statutory regulation or exceeds the permitted use, you will need to obtain permission directly from the copyright holder.
To view a copy of this license, visit https://creativecommons.org/licenses/by/4.0/

Response to Reviewer Comments for

Thermochemical Treatment of Wastewater Residual Solids for Global Mitigation of Emerging Contaminants

REVIEWER 1

1.1. General Comment:

The authors present an exciting approach to using wastewater as a centralized process to remove emerging contaminant from wastewater residual solids (WWRS).

We thank the Reviewer for their supporting comments. We have included point-by-point response to the Reviewer's comments below.

1.2. Main Comment:

1.2.1. *I recommend the authors improve the explanation of why they did not include PFAS in the analysis along with microplastics, PhACs, and antibiotic resistant genes. On page 7, lines 141–142 the authors claim that 90% of PFAS flux into the environment is from industry and yet, we continue to hear about PFAS concentration in WRS and environmental/human exposure to PFAS from soil that was burdened and poisoned with WWRS application, and even airborne exposure from WWRS in landfills (although this could very well be from industrial waste). In addition, on p. 6 lines 102-103 the authors kind of contradict themselves regarding the importance of PFAS in WWRS, when they state that PFOA and PFOS are also concentrated in WWRS compared to the other environmental compartments analyzed in this study. I just think the non -inclusion of PFAS requires more explanation. For example, is it because PFAS requires more extreme conditions for destruction compared to other ECs? Or something else. I'm not saying the authors must include PFAS in the analysis presented in the main manuscript—just please explain better why it's not included.*

We appreciate the Reviewer for this comment. We agree that PFAS concentrations in WWRS are a global concern, consistent with our findings (**Fig. S1**) showing that PFOA and PFOS are enriched in WWRS relative to other environmental compartments. However, the enrichment reflects partitioning behavior rather than the magnitude of global emission pathways. Recent estimates, although highly uncertain, suggest that the overall contribution of WWRS to total PFAS emissions is small (e.g., on the order of 0.1% for PFOA and PFOS[1]). This indicates that PFAS removal within WWRS is unlikely to substantially influence the global PFAS mass balance relative to upstream source-control strategies. This distinction has been clarified in the revised manuscript and has been further supported in **Section S1.5** in the **Supplementary Information**

References:

1. Liu, Z. et al. Pollution pathways and release estimation of perfluorooctane sulfonate (PFOS) and perfluorooctanoic acid (PFOA) in central and eastern China. Science of The Total Environment 580, 1247–1256 (2017).

[Lines 149-152]
Estimates further suggest that contributions routed through WWRS account for only about 0.1% of total emissions of PFOA and PFOS (**Section S1.5**)[61], indicating that PFAS removal within WWRS is unlikely to substantially influence the global PFAS mass balance relative to upstream source-control strategies.

1.3. Minor Comments:

1.3.1. *p. 15, line 297: what is meant by the word indices?*

We have clarified this point by adding examples of the indices used in the text.

[Lines 310-312]
Moreover, while country-level data were employed, cost adjustments were primarily based on indices (e.g., price level index for construction and materials, income-based scaling factors for labor costs) rather than market-based data.

1.3.2. *p. 17 lines 330–331: Please clarify the statement that "Global equity will determine the planetary significance…" It sounds weird. Are you saying that global equity is required. Or that the global north must invest in the global south? Or maybe just what's said in the last sentence of the paragraph that "thermochemical processing must be implemented equitably to restore the chemical balance of the biosphere. " So you could move the last sentence to the start of the paragraph I guess.*

We agree that this wording was unclear. We have revised the sentence to emphasize the importance of global collaboration in achieving equitable implementation.

[Lines 345-346]
Ultimately, achieving a global impact in emerging contaminant mitigation will require equitable implementation across regions.

REVIEWER 2

2.1. General Comment:

The manuscript is a generally well-written overview of emerging contaminants (microplastics, pharmaceuticals, and antibiotic resistant genes) and the use of wastewater residual solids as a removal mechanism. The authors carefully present the ecological and human health concerns surrounding the relatively underregulated release of these classes of emerging contaminants.

We thank the Reviewer for their constructive comments. We have included our responses and revisions below.

2.2. Specific Comments:

The manuscript will be significantly improved with some minor editing and specification of data sources and reference to supplementary data. It was not clear to this reviewer what is the source of data used in creating figures in the main manuscript. It was also difficult to differentiate between figures and tables referenced in the main manuscript and those in the Supplementary Information. Some clarification is needed.

We have clarified the data sources used to generate figures by explicitly referring readers to the **Supplementary Dataset** in each figure caption. We have also clarified in the text that "S" in figure, table, and section numbering indicates **Supplementary Information**.

[At the end of the captions for **Figures 1-3** and **Figures S1-S3**]
Raw data are provided in the **Supplementary Dataset**.

[Lines 103-107]
Complementary analyses of perfluorooctanoic acid (PFOA), perfluorooctane sulfonate (PFOS), and legacy persistent organic pollutants (polybrominated diphenyl ethers, polychlorinated biphenyls, and polychlorinated dibenzo-p-dioxins and polychlorinated dibenzofurans) similarly identify WWRS as highly concentrated global reservoir (**Fig. S1**, where "S" indicates **Supplementary Information**).

REVIEWER 3

3.1. General Comment:

The MS is very interesting, however, I was taken aback by its unconventional organization. Most MSs in scientific lietarure are arranged accorfing to a well-defined layout, helping readers to orientate themselves though the text. In particular, Materials and Methods should come after the introduction, and the section on "Thermochemical technologies for efficient EC destruction" follwoed by results and discussion.

We thank the Reviewer for their constructive suggestion, and we have responded to each individual comment below. Please see responses to **Comment 3.2.5** regarding the layout of the manuscript.

3.2. Specific Comments:

3.2.1. *L. 28 "...ECs—including microplastics (MPs)..." while MPs are certianly relevant as far as detection in many media, a more serious issue is perhaps die to nanoplastics, less detectable and possibly more pernicious (see, e.g. "Micro- and Nano-Plastics in Drinking Water: Threat or Hype? Critical State-of-the-Art Analysis of Risks and Approaches" J Xenob, 2025)*

We have added nanoplastics as an example of ECs in the relevant text and cited the suggested paper.

[Lines 28-32]

ECs—including nanoplastics, microplastics (MPs), pharmaceuticals (PhACs), antibiotic resistance genes (ARGs), and per- and polyfluoroalkyl substances (PFAS)—have been detected across all major environmental compartments, from agricultural soils and river sediments to drinking-water aquifers and oceans[5–21].

3.2.2. *L. 35 "Source-control policies have 35 successfully reduced emissions of well-characterized industrial compounds such as per- and polyfluoroalkyl substances (PFAS)" an interesting discussion was recently published concerning PFAS emissions from WWTPs and the impact of recent regulations ("Prospects of Novel Technologies for PFAS Destruction in Water and Wastewater" Appl Sci, 2025). It appears that some of the observed reductions observed are... just observed due to regulatory loopholes.*

We thank the Reviewer for this comment. While we agree that regulatory factors may influence observed PFAS emission trends, a detailed evaluation of regulatory effectiveness is beyond the scope of this study and not central to this section. Therefore, we have retained the original wording.

3.2.3. *L. 84 "... human-engineered... " pleonastic. Engineered suffices*

We have incorporated this change in the manuscript.

[Lines 86-87]

As the by-product of engineered wastewater treatment systems, WWRS represent a controllable, concentrated, and cross-contaminant point for global ECs elimination.

3.2.4. *L. 129 "...substantial share of total emissions... " I suggest "... substantial share of total waterborne emissions..." (see also the following statement "...20% ... of total global MP emissions migrate through WWRS..."*

We thank the Reviewer for this suggestion. However, the term "total emissions" is intentionally used here because the analysis considers multiple emission pathways beyond waterborne sources (e.g., **Tables S1**, **S3**, and **S5**). We had revised the relevant text to clarify this point.

[Lines 131-132]
Global transmissions modeling of these three ECs reveals that WWRS capture a substantial share of total emissions across environmental pathways (Fig. 1, right panel).

3.2.5. *In addition, I recommend a reision to a more conventional layout before MS can be accepted-*

Based on communication with the editor, the current structure of the manuscript aligns with the requirements of *Nature Communications*.

3.3. Remarks on Code Availability:

I looke dat the code webpage. I was not able to run it, but it seems compliant with general reproducibility requirements.

To further improve usability and reproducibility, we have updated the README file (README.rst) in the GitHub repository to include more detailed instructions for setup and execution. Additionally, we have provided a .yml file (htl_landscape.yml) to facilitate environment configuration and dependency management.
